# Biomimetic hypoxia-triggered RNAi nanomedicine for synergistically mediating chemo/radiotherapy of glioblastoma

**DOI:** 10.1186/s12951-023-01960-w

**Published:** 2023-07-05

**Authors:** Zhen Wang, Xiang-long Tang, Meng-jie Zhao, Yi-ding Zhang, Yong Xiao, Yu-yang Liu, Chun-fa Qian, Yan-dong Xie, Yong Liu, Yuan-jie Zou, Kun Yang, Hong-yi Liu

**Affiliations:** 1 Department of Neurosurgery, The Affiliated Brain Hospital With Nanjing Medical University, Fourth Clinical College of Nanjing Medical University, Nanjing, 210029 China; 2 Department of Neuro-Psychiatric Institute, The Affiliated Brain Hospital With Nanjing Medical University, Nanjing, 210029 China; 3 Institute of Neuro-Science, Nanjing Medical University, Nanjing, 210029 China

**Keywords:** Cancer cell membrane, Hypoxia-triggered, Chemotherapy, Radiotherapy, siRNA delivery, Glioblastoma

## Abstract

Although RNA interference (RNAi) therapy has emerged as a potential tool in cancer therapeutics, the application of RNAi to glioblastoma (GBM) remains a hurdle. Herein, to improve the therapeutic effect of RNAi on GBM, a cancer cell membrane (CCM)-disguised hypoxia-triggered RNAi nanomedicine was developed for short interfering RNA (siRNA) delivery to sensitize cells to chemotherapy and radiotherapy. Our synthesized CCM-disguised RNAi nanomedicine showed prolonged blood circulation, high BBB transcytosis and specific accumulation in GBM sites via homotypic recognition. Disruption and effective anti-GBM agents were triggered in the hypoxic region, leading to efficient tumor suppression by using phosphoglycerate kinase 1 (PGK1) silencing to enhance paclitaxel-induced chemotherapy and sensitize hypoxic GBM cells to ionizing radiation. In summary, a biomimetic intelligent RNAi nanomedicine has been developed for siRNA delivery to synergistically mediate a combined chemo/radiotherapy that presents immune-free and hypoxia-triggered properties with high survival rates for orthotopic GBM treatment.

**Graphical Abstract:**

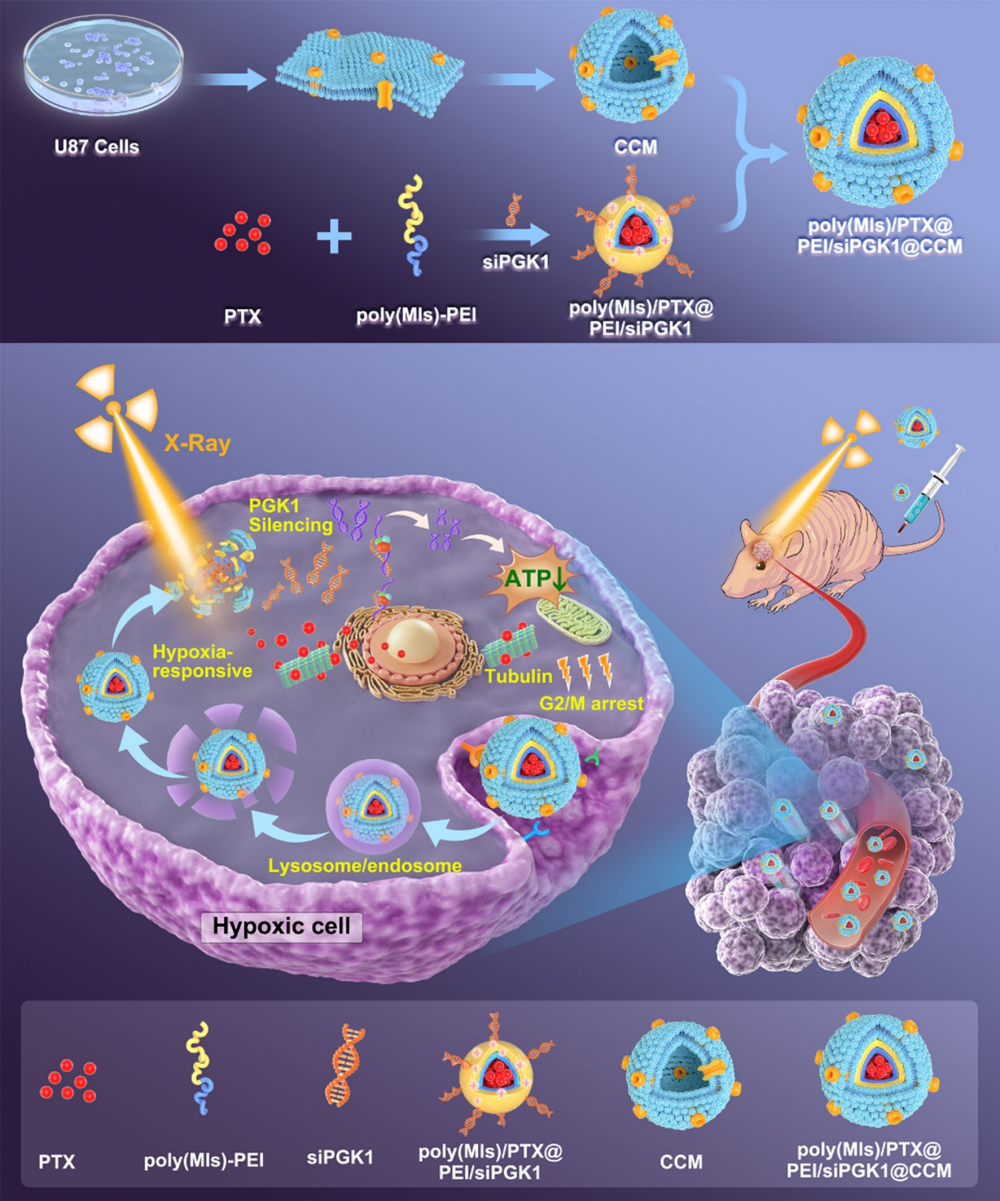

**Supplementary Information:**

The online version contains supplementary material available at 10.1186/s12951-023-01960-w.

## Introduction

Glioblastoma (GBM), a primary malignant and aggressive brain tumor, has a poor prognosis with a low 5-year overall survival rate (< 10%) [1–3]. GBM progression involves multiple gene mutations, and the standard treatment is not very effective in the clinic when ionizing radiation (RT) with concomitant temozolomide (TMZ) is used after maximal surgical resection [4–7]. Therefore, seeking an effective GBM treatment strategy to improve the poor outcomes of current therapeutics is urgently needed.

Increasing evidence has shown that GBM has the ability to initiate chemotherapy and radiotherapy resistance by upregulating the expression of specific genes and promoting cancer cell survival, migration, and tumorigenicity, which inevitably induces tumor recurrence [8–11]. Phosphoglycerate kinase 1 (PGK1) is an adenosine triphosphate (ATP)-generating enzyme and a protein kinase that coordinates energy homeostasis in cellular growth and plays a critical role in regulating cellular activities [12–16]. Intracellular PGK1 overexpression promoted tumor survival by offering adequate ATP in glycolysis process and reprogramming tumor cell metabolism [17–19]. Previous reports suggested that PGK1 protein is upregulated in many types of human cancer, including breast cancer, pancreatic ductal adenocarcinoma and radioresistant astrocytoma [20–22]. Reducing or inhibiting the PGK1 signaling pathway could significantly enhance radiosensitivity and inhibit tumor growth, which is a rational method to improve the outcome of radiotherapy and chemotherapy just by PGK1 silencing [23, 24].

RNA interference (RNAi), a natural cellular process that knocks down the expression of specific genes in cells via short interfering RNA (siRNA), has been considered a highly promising strategy for GBM treatment [25–27]. However, this method was still confined to the laboratory and is limited by siRNA delivery efficiency caused by rapid degradation and clearance from the circulating bloodstream [28–30]. Thus, the development of a new highly efficient siRNA delivery system is highly desirable. In the past decade, nonviral vectors have been developed to increase siRNA circulation time in vivo by avoiding the recognition of reticuloendothelial systems (RESs), such as the liver and spleen. To achieve tumor-specific siRNA delivery, many delivery systems have been designed on the basis of the well-known enhanced permeability and retention (EPR) effect, tumor-specific receptor recognition and intelligent responsiveness to the unique tumor microenvironment (TME) [31–36]. Recently, biomimetic cancer cell membrane (CCM)-cloaking nanosystems have exhibited the ability to maintain innate immune escape ability, physical barrier-crossing ability, and homotypic recognition and have been extensively studied [37–41]. CCM coating presented specific adhesive to homotypic tumor via self-recognition of cancer cells and “homing” to the homologous tumor. Therefore, exploring effective siRNA delivery systems with biomimetic CCM shells is an appropriate approach and would undoubtedly be beneficial to improving the RNAi therapeutic effect on GBM.

**Fig. 1:**
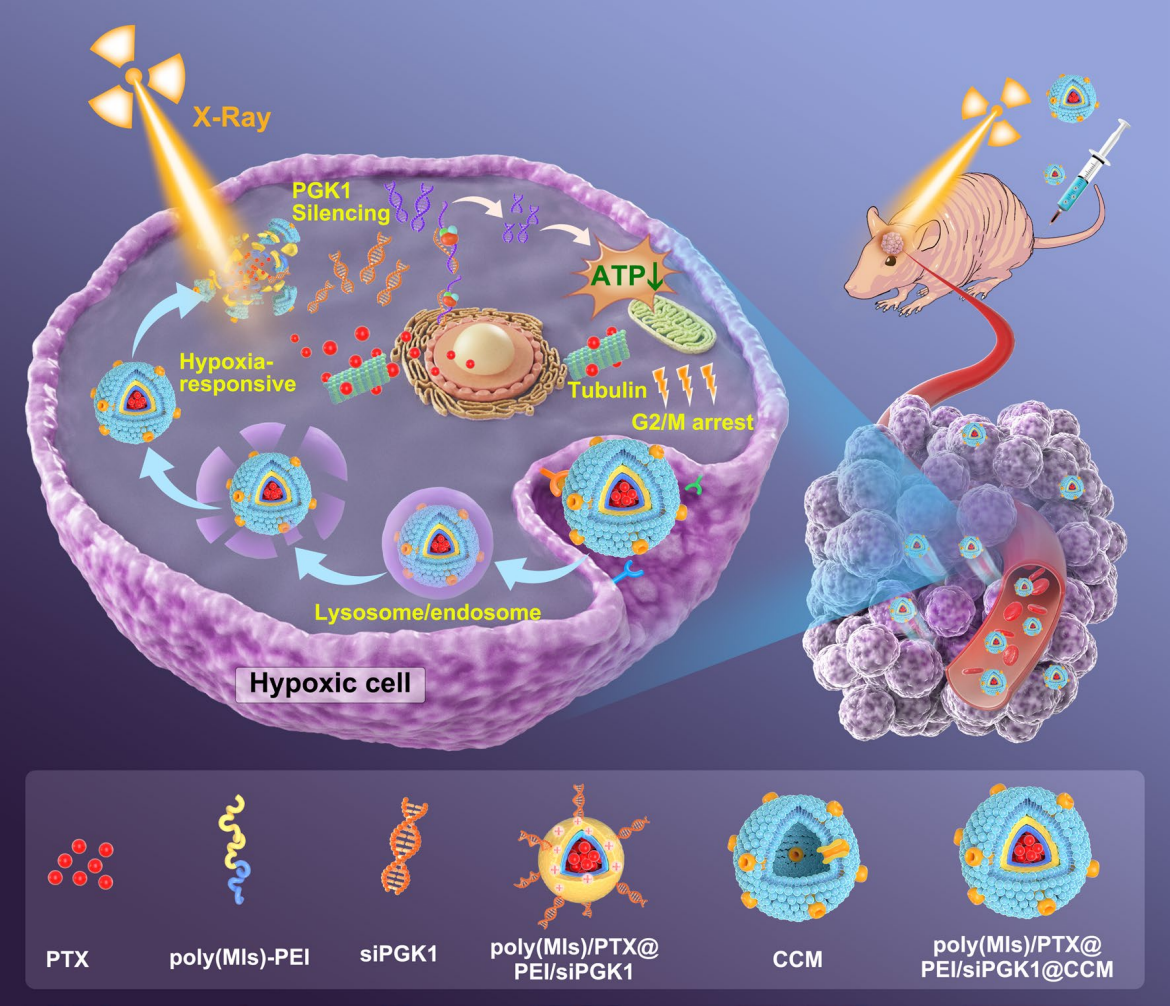
Schematic mechanism of hypoxia-triggered RNAi nanomedicine for synergistically sensitizing orthotopic glioblastoma to chemo/radiotherapy by PGK1 silencing

In this study, we constructed a biomimetic hypoxia-triggered RNAi nanomedicine (poly(MIs)/PTX@PEI/siPGK1@CCM) for synergistically sensitizing chemotherapy and radiotherapy by inhibiting PGK1-promoted GBM progression. Hypoxia-triggered amphiphilic poly(MIs)-PEI block polymers were successfully synthesized and designed as inner cores by paclitaxel (PTX)-induced self-assembly into hydrophobic poly(MIs) domains. Negative charged siPGK1 was condensed on the outer PEI backbone by electrostatic interaction and further protected by the CCM layer against blood enzymes and adverse immune responses in plasma. Our biomimetic CCM-disguised RNAi nanomedicine showed an excellent ability to hone in brain tumors by homotypic adhesive interactions that were verified in orthotopic U87MG tumor-bearing nude mice. The efficient endosomal/lysosomal escape of poly(MIs)/PTX@PEI/siPGK1@CCM by the proton sponge effects derived from the cationic poly(MIs)-PEI polymer and then hypoxia-triggered structural conversion that hydrophobic poly(MIs-NO_2_) chain was converted into hydrophilic poly(MIs-NH_2_) by bioreductions in hypoxic TME, which led to structure dissociation and the release of encapsulated PTX and siPGK1 (Fig. 1). The chemical agent PTX enhanced tubulin polymerization and stabilized microtubules that blocked GBM cell division and arrested the cell cycle at G2/M to sensitize DNA damage induced by ionizing radiation. Moreover, down-regulation of PGK1 protein efficiently inhibited cellular energy supply and exhibited sensitive behavior in synchronous chemo/radiotherapy. Generally, we provided a new modality that depended on PGK1 knockdown, which synergistically enhanced chemo/radiotherapy and showed an excellent antitumor effect both in vitro and in vivo.

## Experiment section

### Materials

Metronidazole (MI), 5-benzyl-l-glutamate-n-carboxy-anhydride (Boc-Glu-NCA) and poly(ethylenimine) (PEI, MW = 2 kDa) were purchased from Xian Ruixi Biotechnology Company. Palitaxel (PTX), Cell Counting Kit-8 (CCK-8) and Doxorubicin (DOX) were purchased from Sigma-Aldrich. U87MG-Luc cells were established by lentivirus transfection in our lab. A live/dead kit, EdU proliferation test kit and annexin V-FITC apoptosis detection kit were purchased from Beyotime. PGK1 and Ki67 antibodies were purchased from Abcam, etc. The sequences of siPGK1 were as follows: sense strand, 5ʹ-CCA AGU CGG UAG UCC UUA UTT-3ʹ; antisense stand, 5′-AUA AGG ACU ACC GAC UUG GTT-3ʹ. FAM dye was linked to the 5′-end of the antisense strand. All other solvents and reagents were of analytical grade.

### Material characterization

The morphological changes were characterized by transmission electron microscopy (TEM; JEOL JEM-1010). Dynamic light scattering measurements and zeta potential analysis were determined at 25 °C by a Zetasizer Nano ZS instrument (Malvern). FT-IR spectroscopy (Nexus 670, Nicolet, USA) was recorded by KBr sample holder method. The cell membrane fragments on poly(MIs)/PTX@PEI/siPGK1@CCM were determined through SDS-PAGE protein analysis.

### poly(MIs)-PEI block copolymer synthesis

In brief, poly(MIs)-PEI block copolymers were synthesized in four steps: (1) Boc-Glu-NCA was added to chloroform with slow stirring, and 4-aminobutyric acid was subsequently added at a molar ratio of 42:1. When the solution was changed to colorless, diethyl ether was used to precipitate the poly(Boc-Glu-NCA) product. (2) The purified white poly(Boc-Glu-NCA) polymer was dissolved in 33% HBr/AcOH solution, and a certain volume of CF_3_COOH (v/v = 5:1) was slowly added. After the solution proceeded for 2 h, an excess amount of diethyl ether was poured, and the yellow poly(Glu-COOH) precipitate was washed three times. (3) The synthesized poly(Glu-COOH) polymer was dissolved in DMF solution; EDCI, DMAP and MI were added; and the mixture was stirred for 24 h at room temperature. poly(Glu-COOH), EDCI, 4-dimethylaminopyridine (DMAP) and MI were optimized at a molar ratio of 0.5:25:5:21. The crude product poly(Glu-MIs) was obtained after purification. The Mw was determined to be ~ 1580 by gel permeation chromatography (GPC). (4) To obtain a poly(MIs)-PEI copolymer, the polymer poly(Glu-MIs) was dissolved in DMF, and a certain amount of EDCI, DMAP and branched PEI (MW, 2 kDa) was added in a molar ratio of 1:1.2:0.4:1. Then, the solution was mixed and stirred at room temperature for 24 h. After the reaction, dialysis was performed in deionized water (5 kDa) for 72 h (the dialysis fluid was changed every 8 h). The poly(MIs)-PEI polymer was obtained by freeze drying and quantitatively characterized via ^1^H NMR (300 MHz, D_2_O/DMSO-d6; ppm). The Mw of poly(MIs)-PEI copolymer was mainly centered at 3800 determined by GPC (Agilent 1260).

### Preparation of CCM vesicles

The U87MG cell membranes were prepared according to a previous report [39]. In brief, U87MG cells were detached and washed 3 times after centrifugation for 5 min (600 g). To harvest the membranes, the cells were suspended in hypotonic lysis buffer (20 mM Tris–HCl, 10 mM KCl, 2 mM MgCl_2_ and protease inhibitor) and disrupted by a Dounce homogenizer. The supernatants were obtained by centrifugation for 10 min at 700 g and were pooled and centrifuged for 30 min (14,000*g*). The pellet containing the plasma membrane material was then washed and collected as purified cancer cell membranes.

### Preparation of poly(MIs)/PTX@PEI/siPGK1@CCM nanomedicine

poly(MIs)/PTX@PEI/siPGK1@CCM was prepared by the following protocol: In brief, 50 mg of yellow poly(MIs)-PEI powder was dissolved in 5 mL of DMSO with slow stirring at 60 °C for 30 min. Then, the yellow solution cooled to room temperature, and PTX (4.2 mg) dissolved in DMSO (0.5 mL) was added followed by continued stirring for 1 h. poly(MIs)/PTX@PEI was obtained by dialysis against a PBS solution for 24 h. siPGK1 (2 OD, 66 μg) was dissolved in 200 μL RNase-free water, added to poly(MIs)/PTX@PEI solution (1.9 mg/mL) and stirred at room temperature for 15 min. After that, the free siPGK1 was removed by ultrafiltration (Mw: 8 kDa). Finally, to coat cancer cell membranes onto poly(MIs)/PTX@PEI/siPGK1, 1 mL of freshly synthesized poly(MIs)/PTX@PEI/siPGK1 solution was mixed with the purified CCM, and the mixture was subsequently extruded through 800 nm, 400 nm and 200 nm pores. The excess uncoated CCM was removed by a Sephadex G-200 column. The loading and encapsulation efficiency of PTX were calculated by the following Eqs. (1) and (2). The loading and encapsulation efficiency of siPGK1 was calculated as the similar procedure.

1 $$\mathrm{PTX \,loading \,efficicency \%}=\frac{\mathrm{m}\left(\mathrm{PTX}\right)-\mathrm{m}\left(\mathrm{PTX \,in \,solution}\right)}{\mathrm{total \,m}\left(\mathrm{poly}\left(\mathrm{MIs}\right)/\mathrm{PTX}@\mathrm{PEI}/\mathrm{siPGK}1@\mathrm{CCM}\right)}*100$$

2 $$\mathrm{PTX \,encapsulation \,efficicency \%}=\frac{\mathrm{m}\left(\mathrm{PTX}\right)-\mathrm{m}\left(\mathrm{PTX \,in \,solution}\right)}{\mathrm{m}\left(\mathrm{PTX}\right)}*100$$

### Gel retardation assays

The protective effects of poly(MIs)/PTX@PEI/siPGK1@CCM against external enzyme degradation and hypoxia-triggered release behavior of siPGK1 were studied via agarose gel electrophoresis. Poly(MIs)/PTX@PEI/siPGK1@CCM (equivalent siPGK1, ~ 1.2 mM) or NADPH-treated poly(MIs)/PTX@PEI/siPGK1@CCM with different time period (10, 20, 40, 60 min) was added to HEPES buffer solution and then incubated for 30 min. The incubated mixture was electrophoresed through a 2% agarose gel (110 V, 10 min). The gel images were captured by an imaging system (Bio-Rad Gel Doc™ XR).

### Cell cytotoxicity assays

The biocompatibility of poly(MIs)@PEI/@CCM and the in vitro cytotoxicity of poly(MIs)/PTX@PEI/siPGK1@CCM on U87MG cells were investigated by CCK-8 assay. In general, U87MG cells were plated in 96-well plates and cultured under normoxic (21% O_2_, 5% CO_2_ and 74% N_2_) or hypoxic conditions (1% O_2_, 5% CO_2_ and 94% N_2_) overnight. Various concentrations of poly(MIs)@PEI/@CCM and poly(MIs)/PTX@PEI/siPGK1@CCM (100, 200, 300, 400, 500, 800 and 1000 μg/mL) were added and incubated with U87MG cells for another 24 h. All treated U87MG cells were incubated with CCK-8 PBS solution for 4 h. The proportion of cell viability was calculated after the absorbance values of the cells were determined with a microplate reader at 450 nm.

### In vitro BBB transcytosis

Mouse bEnd.3 brain endothelial cells were plated on a transwell insert to form a compact monolayer in the upper chamber, and the value of transepithelial electrical resistance (TEER) was measured and remained > 200 Ω cm^2^ throughout our study, as detected by a Millicell®-ERS-2 (Millipore) instrument. Free FAM-siPGK1 and poly(MIs)/PTX@PEI/siPGK1@CCM were added to each upper chamber, and BBB transcytosis ability at different time points (2 h, 4 h and 6 h) was evaluated by the fluorescence intensity of siPGK1 in the supernatant of basolateral medium.

### Cellular uptake

To monitor poly(MIs)/PTX@PEI/siPGK1@CCM uptake behavior by U87MG cells, U87MG cells were seeded at a density of 1 × 10^5^ cells/well and cultured overnight. Then, U87MG cells were pretreated with FAM-labeled poly(MIs)/PTX@PEI/siPGK1@CCM for a certain time. Cell uptake and lysosome/endosome escape were detected by FAM fluorescence per cell using a confocal laser scanning microscope (CLSM). The intracellular hypoxia-responsive release behavior was investigated by determining the DOX red fluorescence location of poly(MIs)/DOX@PEI/siPGK1@CCM. Generally, U87MG cells were cultured in CLSM culture dishes under hypoxic or normoxic conditions overnight. Poly(MIs)/DOX@PEI/siPGK1@CCM (150 μg/mL) was added and incubated for 4 h. siPGK1 release behavior from poly(MIs)/PTX@PEI/siPGK1@CCM was captured by a CLSM imaging system.

### Cell cycle analysis

U87MG cells were treated for 24 h with poly(MIs)/PTX@PEI/siPGK1@CCM (1 μM PTX) in 6-well plates. The cells were then fixed with 70% EtOH and washed several times. The cells were subsequently stained with PI (50 μg/mL) for 30 min and analyzed by flow cytometry (FACSCalibur).

### Live/dead cell staining assays

U87MG cells were cultured in 6-well plates at a density of 1 × 10^5^ cells/well, and the cells were treated with biomimetic poly(MIs)/PTX@PEI/siPGK1@CCM at different concentrations (2*10^–6^ M, 4*10^–6^ M and 6*10^–6^ M). After incubation for 4 h, the U87MG cells were washed with PBS several times. Afterward, these treated cells were costained with calcein AM and propidium iodide (PI) for 30 min and washed with PBS several times. Finally, the cells were analyzed with a fluorescence microscope.

### Colony formation assays

Colony formation of different groups was performed as previously described [42]. In brief, U87MG cells were seeded in 6-well plates and then treated with poly(MIs)/PTX@PEI/siPGK1@CCM (0.15 mg/mL) for 24 h. Afterward, the 6-well plates were exposed to different radiation dosages (0, 2, 4, 6 and 8 Gy). The culture medium was discarded after radiation for 1 h, and fresh DMEM was added and incubated for two weeks. Cell clones were fixed with 4% paraformaldehyde and stained with crystal violet after 2 weeks. All colonies consisting of 50 or more cells were counted.

### EdU assays

EdU assays were carried out after U87MG cells were treated with different formulations. The treated U87MG cells were cultured with 50 μM 5-ethynyl-2’-deoxyuridine (EdU) medium for 2 h and washed with PBS several times. Then, the cells were fixed with 4% formaldehyde and permeabilized with 0.5% Triton X-100. The proliferation of U87MG cells was visualized using Apollo®643. Hoechst counterstaining was performed to identify cell nuclei.

### Cell immunofluorescence for H2AX

U87MG cells were cultured in a 12-well plate overnight and treated with free PTX and poly(MIs)/PTX@PEI/siPGK1@CCM for 4 h under hypoxic conditions (21% O_2_, 5% CO_2_ and 74% N_2_) and under normoxic conditions. Cells were irradiated (2 Gy) and stained with a γH2AX antibody (red) after irradiation. The cells were then analyzed using fluorescence microscopy and imaged.

### Apoptosis analysis by flow cytometry

The cell death efficiency and mechanism of siPGK1-mediated chemo/radiotherapy of poly(MIs)/PTX@PEI/siPGK1@CCM was investigated by typical flow cytometry apoptosis. Normoxic and hypoxic U87MG cells were seeded in 6-well plates for 24 h and then treated with saline, free PTX + siPGK1, poly(MIs)@PEI/siPGK1@CCM, poly(MIs)/PTX@PEI@CCM and poly(MIs)/PTX@PEI/siPGK1@CCM. After 4 h, these treated cells were exposed to 6 Gy RT. Finally, cell apoptosis was detected using an Annexin V-FITC/PI apoptosis detection kit analyzed by flow cytometry (FACSCalibur) in accordance with the manufacturer’s protocol.

### Animal model

Nude mice (female, 16–20 g) received care in accordance with the guidelines of the Institutional Animal Care and Use Committee (IACUC) of the Ethics Committee of Nanjing Medical University (IACUC-1902028). Five microliters of 1 × 10^5^ U87MG-Luc cells suspended in serum-free media was injected into the brain region, and U87MG glioblastoma orthotopic xenografts were prepared after 10 days and assessed via an animal imaging system.

### In vivo biodistribution of poly(MIs)/PTX@PEI/siPGK1@CCM

1. A single dose of poly(MIs)/PTX@PEI/siPGK1@LipoPEG, poly(MIs)/PTX@PEI/siPGK1@CCM and free siRNA in 200 μL PBS was administered intravenously via the tail vein (2.3 mg FAM-siRNA equiv./kg) into U87MG-luc glioblastoma orthotopic tumor-bearing nude mice. At different time points (0.2, 0.5, 1, 2, 4, 6, 8, 10, 12 h), 50 μL blood samples from the eye socket was lysed in Triton X-100 assisted with sonication for 15 min and the FAM-siPGK1 concentration in supernatant was determined by a microplate reader (Ex = 494 nm, Em = 522 nm) after centrifugation (15,000 rpm, 5 min).
2. A single dose of three different formulations (2.3 mg FAM-siRNA equiv./kg) was administered intravenously into glioblastoma-bearing nude mice. In vivo distribution of fluorescence images was acquired with a Lumina IVIS III near-infrared fluorescence imaging system at different time points (2, 4, 8, 12, 24 h).
3. Nude mice were divided into three groups and received the corresponding treatment (2.3 mg FAM-siRNA equiv./kg). At different time points (0, 2, 4, 8, 12, 24 h), the mice was dissected and major organs (brain, heart, liver, spleen, lung and kidney) were collected for determining FAM-siRNA level. The brain tissues were further fixed in 4% paraformaldehyde for 24 h and dehydrated in gradient sucrose gradient dehydration. Frozen sections of 5 μm thickness were prepared and stained with DAPI for 15 min and then photographed by fluorescence microscopy.

### In vivo antitumor efficiency

The nude mice bearing U87MG glioblastoma cells were weighed and randomly divided into seven groups (n = 10): saline (group 1), saline + RT (group 2), free PTX + naked siPGK1 + RT (group 3), poly(MIs)/PTX@PEI/siPGK1@CCM (without RT, group 4), poly(MIs)@ PEI/siPGK1@CCM + RT (group 5), poly(MIs)/PTX@PEI@CCM + RT (group 6), poly(MIs)/PTX@PEI/siPGK1@CCM + RT (group 7), and received the different treatments via the tail vein every 2 days. The PTX and siPGK1 doses were ~ 1.8 mg/kg and ~ 2.3 mg/kg, respectively. Whole-brain radiation therapy (2 Gy, 0.2 Gy/min) was performed via X-ray equipment. Tumor inhibition in all groups was evaluated by live bioluminescence imaging at different time points captured by a Maestro animal imaging system. The relative tumor size was determined using the following formulas: Relative tumor size (Vt/V_0_) = bioluminescence intensity at day t (V_t_) /bioluminescence intensity at day 10 (V_0_). In the whole study, mice were weighed every 2 days, and Kaplan–Meier survival curves were generated for each group.

### Histological examination

All nude mice were sacrificed to evaluate in vivo toxicity after different treatment strategies. The mice were dissected to isolate the tumor, heart, liver, spleen, lung, and kidneys and fixed in 4% formalin. All sliced organs were stained with hematoxylin and eosin (H&E) and was observed by changes in their features. The GBM tumor tissues in the brain were pretreated with Triton X-100 (0.1%) and BSA serum (5%). For molecular pathology assessment, these slices were incubated overnight together with PGK1, Ki67 and rH2AX antibodies at 4 ºC. For DNA injury examination, a TdT-mediated dUTP nick end labeling (TUNEL) assay was processed and costained with cell nuclei (DAPI).

### Blood hemanalysis and biochemical analyses

Blood samples were collected from healthy mice after poly(MIs)/PTX@PEI/siPGK1@CCM (PTX: ~ 1.8 mg/kg, siPGK1: ~ 2.3 mg/kg) was administered intravenously to tumor-free mice (n = 3) for 24 h. Hemanalysis and biochemical analyses were performed to analyze ALT, AST, TBIL, BUN, CREA, Hbg, Plt, RBC and WBC levels.

### Statistical analysis

The experimental data are expressed as the means ± SDs. The comparison of two groups was performed by Student’s t-test. Survival results were analyzed by the Kaplan–Meier technique using GraphPad Prism software. *p < 0.05 was considered significant; **p < 0.01 and ***p < 0.001 were considered highly significant.

## Results and discussion

### Synthesis and characterization

**Fig. 2:**
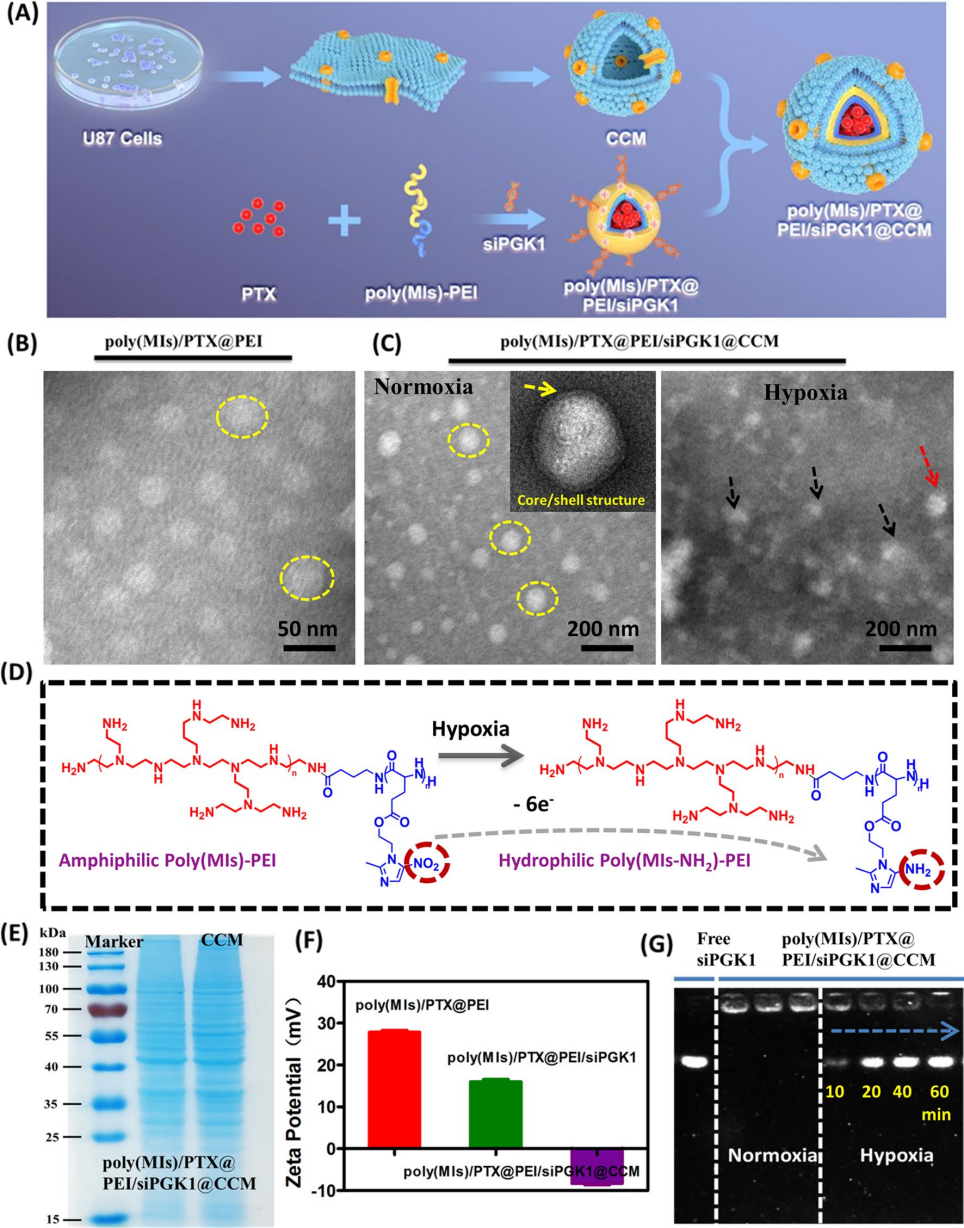
**A** Schematic design of the synthesis of biomimetic hypoxia-triggered poly(MIs)/PTX@PEI/siPGK1@CCM. **B** TEM morphology of poly(MIs)/PTX@PEI. **C** TEM morphology of poly(MIs)/PTX@PEI/siPGK1@CCM under normoxia and hypoxia. Inset: core/shell structure by TEM in high-resolution and yellow arrow indicated CCM shell. **D** The structural transformation mechanism of amphiphilic poly(MIs)-PEI into hydrophilic poly(MIs-NH_2_)-PEI by reduction of nitro (−NO_2_) to amine (−NH_2_) under hypoxic conditions catalyzed by intracellular nitroreductases. **E** Membrane protein visualization of poly(MIs)/PTX@PEI /siPGK1@CCM and the whole cancer cell membrane (CCM) through SDS-PAGE analysis. **F** Zeta potential changes of the PBS solution. **G** siPGK1 release behavior of poly(MIs)/PTX@PEI/siPGK1@CCM under hypoxia and normoxia (10, 20, 40, 60 min)

In this study, biomimetic poly(MIs)/PTX@PEI/siPGK1@CCM was designed and successfully prepared by the following procedure (Fig. 2A). First, a poly(MIs) polymer was synthesized by ring-opening polymerization of the Boc-Glu-NCA monomer and the esterification process of poly(Glu-COOH) with MI (Additional file 1: Fig. S1). The amphiphilic cationic block copolymer poly(MIs)-PEI was obtained by the acylation reaction of poly(MIs) with branched PEI and characterized by ^1^H NMR (Additional file 1: Fig. S2), which presented typical chemical shift of metronidazole (MI) and PEI. Strong adsorption peaks observed at 1630 cm^−1^ by FT-IR spectra further demonstrated the amide linkage (Additional file 1: Fig. S3). Then, PTX, a clinical chemical antitumor drug, was loaded into the hydrophobic core to obtain poly(MIs)/PTX@PEI by hydrophobic interactions between PTX molecules and the poly(MIs) chain. Finally, siPGK1 was adsorbed on the PEI backbone, and freshly extractive CCM was immediately coated on the surface to fabricate poly(MIs)/PTX@PEI/siPGK1@CCM by the coextrusion of poly(MIs)/PTX@PEI/siPGK1 and CCM. The loading efficiency of siPGK1 and PTX was 3.66 ± 0.19% and 7.35 ± 0.28%, respectively. The encapsulation efficiency of siPGK1 and PTX was about ~ 96% and ~ 89.2%, respectively. The well-defined spherical morphology with uniform size of synthesized poly(MIs)/PTX@PEI had been successfully demonstrated via transmission electron microscopy (TEM) imaging (Fig. 2B), and the average hydrodynamic diameter in PBS solution was determined at 55 nm with a narrow size distribution, as determined by dynamic light scattering (DLS) measurements (Additional file 1: Fig. S4). Moreover, interestingly, after siPGK1 was tightly condensed on the backbone of poly(MIs)/PTX@PEI by the electrostatic interaction between positively charged PEI and negatively charged siPGK1, the size decreased by ~ 10 nm compared with that of poly(MIs)/PTX@PEI. The reason was attributed to the charge decrease of PEI by adsorption of genes in PBS, which reduced hydration effect and presented a tight composite structure. By adjusting the ratio of various components, we determined that the hydrodynamic diameter of synthesized poly(MIs)/PTX@PEI/siPGK1@CCM centered at 107 nm with a typical core/shell structure (a thin CCM shell) had a narrow polydispersity index, which was consistent with the TEM morphology (Fig. 2C). To further confirm the hypoxia-trigged change in poly(MIs)/PTX@PEI/siPGK1@CCM morphology, poly(MIs)/PTX@PEI/siPGK1@CCM were incubated under hypoxic and normoxic conditions. Interestingly, compared to the normoxic condition, the structure of poly(MIs)/PTX@PEI/siPGK1@CCM rapidly swollen at the initiating time and collapsed within 4 h under hypoxic conditions (Additional file 1: Figure S5A), which attributed to the polymer structure change from hydrophobic poly(MIs-NO_2_) chain to hydrophilic poly(MIs-NH_2_) chain catalyzed by NADPH enzymes (Fig. 2D) that was verified by the adsorption peak shift from 318 to 338 nm in UV–vis absorption spectrum (Additional file 1: Fig. S5B). The stability of poly(MIs)@PEI@CCM, poly(MIs)/PTX@PEI@CCM, poly(MIs)@PEI/siPGK1@CCM and poly(MIs)/PTX@PEI/siPGK1@CCM were also investigated in DMEM containing 10% FBS. No significant size increases were observed in these formulations over seven days (Additional file 1: Fig. S6). These results suggested that poly(MIs)@PEI@CCM, poly(MIs)/PTX@PEI@CCM, poly(MIs)@PEI/siPGK1@CCM and poly(MIs)/PTX@PEI/siPGK1@CCM had a good colloidal stability in 10% FBS because of the negative surface charge that enhanced colloidal stability and prolonged colloidal residence time by reducing protein adsorption and aggregation. To further confirm the stability of poly(MIs)/PTX@PEI/siPGK1@CCM in a physiological environment, the zeta potential was detected. After CCM camouflaging, the surface charge of poly(MIs)/PTX@PEI/siPGK1 was changed from positive to negative (Fig. 2F), which indicated that CCM was successfully modified on the surface and further directly verified by western blot analysis, which retained most membrane protein compositions (Fig. 2E). The hypoxia-triggered siPGK1 release behavior of poly(MIs)/PTX@PEI/siPGK1@CCM was assessed by the gel retardation assay (Fig. 2G). As shown, the entrapped siPGK1 demonstrated limited release from poly(MIs)/PTX@PEI/siPGK1@CCM under normoxia and presented an hypoxia-triggered siPGK1 release under hypoxia, indicating an effective protection of siPGK1 from degradation by RNase when it was circulated in bloodstream. We further predicated that poly(MIs)/PTX@PEI/siPGK1@CCM have great potential for hypoxia-responsive controlled drug release. Thus, the release property of both PTX and siPGK1 from poly(MIs)/PTX@PEI/siPGK1@CCM was investigated under both hypoxic and normoxic conditions at pH 7.4 PBS and 37 °C. However, less than 20% drug could be released in 120 h, indicating that poly(MIs)/PTX@PEI/siPGK1@CCM could be stable in the blood circulation and in the extracellular environment of tumor tissues (Additional file 1: Fig. S7, orange and green squares). While under hypoxic conditions, the PTX cumulative release rate reached 81% and 92% within 24 h and 120 h, respectively (Additional file 1: Fig. S7, red squares). This phenomenon was attributed to that poly(MIs)/PTX@PEI/siPGK1@CCM selectively dissociated and released PTX under hypoxia. The release behavior of siPGK1 was similar but slightly slower than that of PTX, suggesting that poly(MIs-NH_2_)-PEI/siPGK1 successfully dissociated from poly(MIs)/PTX@PEI/siPGK1@CCM and was fully exposed to acidic environment, which triggered the effective siPGK1 release from PEI backbone due to the H^+^-siPGK1 interaction that decreased negative density on siPGK1 surface and reduced siRNA condensing on PEI (Additional file 1: Fig. S6, blue squares). Thus, PTX and siPGK1 would be rapidly released inside acidic tumor instead of other normal tissue. Although the CCM coating could protect siRNAs from degradation by external serum nucleases, it enabled small molecules, including ethidium bromide, to penetrate into the inner and stain siPGK1. To mimic bioreductions in hypoxia, poly(MIs)/PTX@PEI/siPGK1@CCM was previously treated with NADPH enzyme for different time (10, 20, 40, 60 min), and it was found that siPGK1 could be efficiently released from CCM protection and presented a time-dependent release behavior, demonstrating highly effectively hypoxia-responsive release properties.

### Evaluation of the BBB permeation ability

**Fig. 3:**
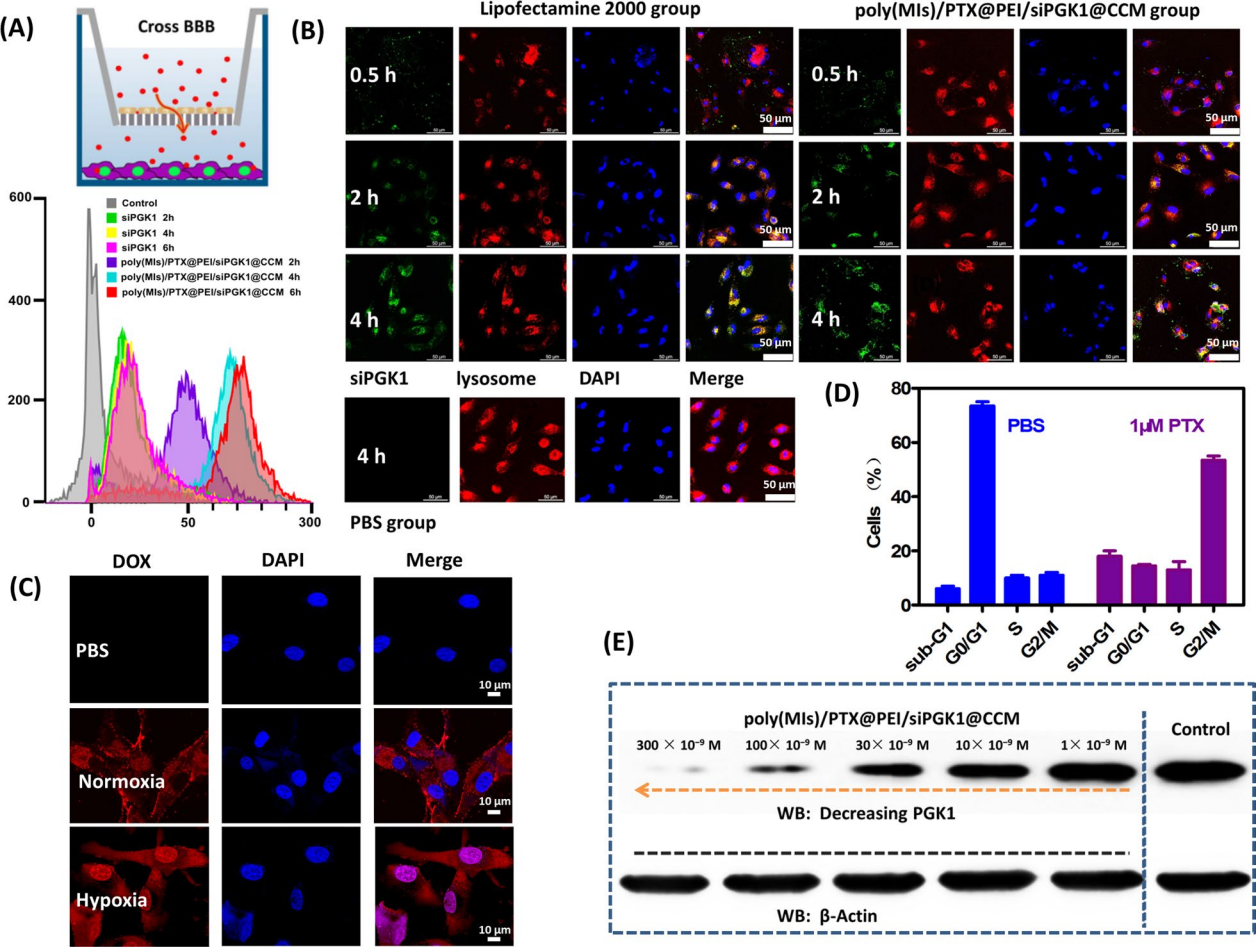
**A** Cumulative transport of poly(MIs)/PTX@PEI/siPGK1@CCM at different time points across the bEnd.3 monolayer. Inset: schematic illustration of BBB model. **B** CLSM images of U87 cells incubated with commercial Lipofectamine 2000 and poly(MIs)/PTX@PEI/siPGK1@CCM for different times. Green fluorescence shows location of the FAM-siPGK1. The lysosomes/endosomes were stained with LysoTracker Red (red), and the cell nuclei were stained with DAPI (blue). **C** The cellular uptake and release behavior after incubation for 4 h in hypoxic and normoxic conditions. **D** The percentages of each cell cycle subpopulation in poly(MIs)/PTX@PEI/siPGK1@CCM-treated U87 cells. **E** PGK1 protein inhibition capacity after 24 h of transportation

To evaluate blood brain barrier (BBB) permeation of poly(MIs)/PTX@PEI/siPGK1@CCM, the cumulative transport across the BBB layer was investigated by an in vitro transwell model (Fig. 3A). The FAM-siPGK1 concentration on the basolateral side was measured at different times by a flow cytometry fluorescence, and a time-dependent BBB permeation phenomenon was observed. Quantitative analysis illustrated that free siPGK1 hardly crosses the BBB layer (Additional file 1: Fig. S8), but poly(MIs)/PTX@PEI/siPGK1@CCM could cross the BBB model to arrive at the basolateral side more efficiently and 20-fold higher than free siPGK1. This phenomenon indicated that poly(MIs)/PTX@PEI/siPGK1@CCM was an effective vector to enhance the transport of the siRNA across the BBB, which was attributed to biomimetic CCM cloaking.

### Evaluation of lysosomal escape and anti-agent release

The intracellular trafficking and release behavior of poly(MIs)/PTX@PEI/siPGK1@CCM was investigated by confocal laser scanning microscopy. To monitor endosomal/lysosomal escape, the lysosomes of U87MG cells were stained with LysoTracker Red. Obviously, a significant amount of poly(MIs)/PTX@PEI/siPGK1@CCM was efficiently internalized and trapped in lysosomes/endosomes following 2 h incubation with U87MG cells (Fig. 3B). In contrast, a stronger fluorescence signal was observed in the cytoplasm after 4 h of incubation. This phenomenon indicated that the poly(MIs)/PTX@PEI/siPGK1@CCM nanomedicine had a good lysosomal escape ability, which contributed to the inner PEI structure with the unsaturated amino group that disrupted endosomes/lysosomes by the proton sponge effect [43, 44]. After the establishment of the endosomal/lysosomal escape pathway, hypoxia-responsive release was further investigated by doxorubicin (DOX)-loaded poly(MIs)/DOX@PEI/siPGK1@CCM after 4 h of incubation (Fig. 3C). Considering that free DOX is easily transported to the nucleus and provides more convenience to observe the hypoxia-triggered release behavior, antitumor PTX molecules were replaced with DOX. Notably, the location of DOX was mainly detected in the cytoplasm under normoxic conditions. Conversely, under hypoxic conditions, the DOX molecules were transported to the nucleus and showed strong red fluorescence, demonstrating the efficient hypoxia-induced decomposition of our RNAi nanomedicine, which played a vital role in siRNA intracellular release. PTX release on the cell cycle of U87MG cells was further verified by measuring PI-stained cells using flow cytometry. The untreated U87MG cells showed ~ 12% of all cells at G2/M phase of the cell cycle (Fig. 3D). In contrast, poly(MIs)/PTX@PEI/siPGK1@CCM-treated U87MG cells showed 58% G2/M arrest, which indicated that PTX induced G2/M arrest in the U87MG cell line with a parallel decrease in the G0/G1 population. To evaluate gene-silencing effect of poly(MIs)/PTX@PEI/siPGK1@CCM-based RNAi therapy, transfection assays were carried out using western blot analysis **(**Fig. 3E), and the results indicated that our RNAi nanomedicine induced significant down-regulation of PGK1 gene expression under different siPGK1 concentrations. PGK1 protein levels were downregulated up to 83% by poly(MIs)/PTX@PEI/siPGK1@CCM at the maximal siRNA concentration of 300 × 10^−9^ M after 24 h of transfection.

### Evaluation of cellular apoptosis

**Fig. 4:**
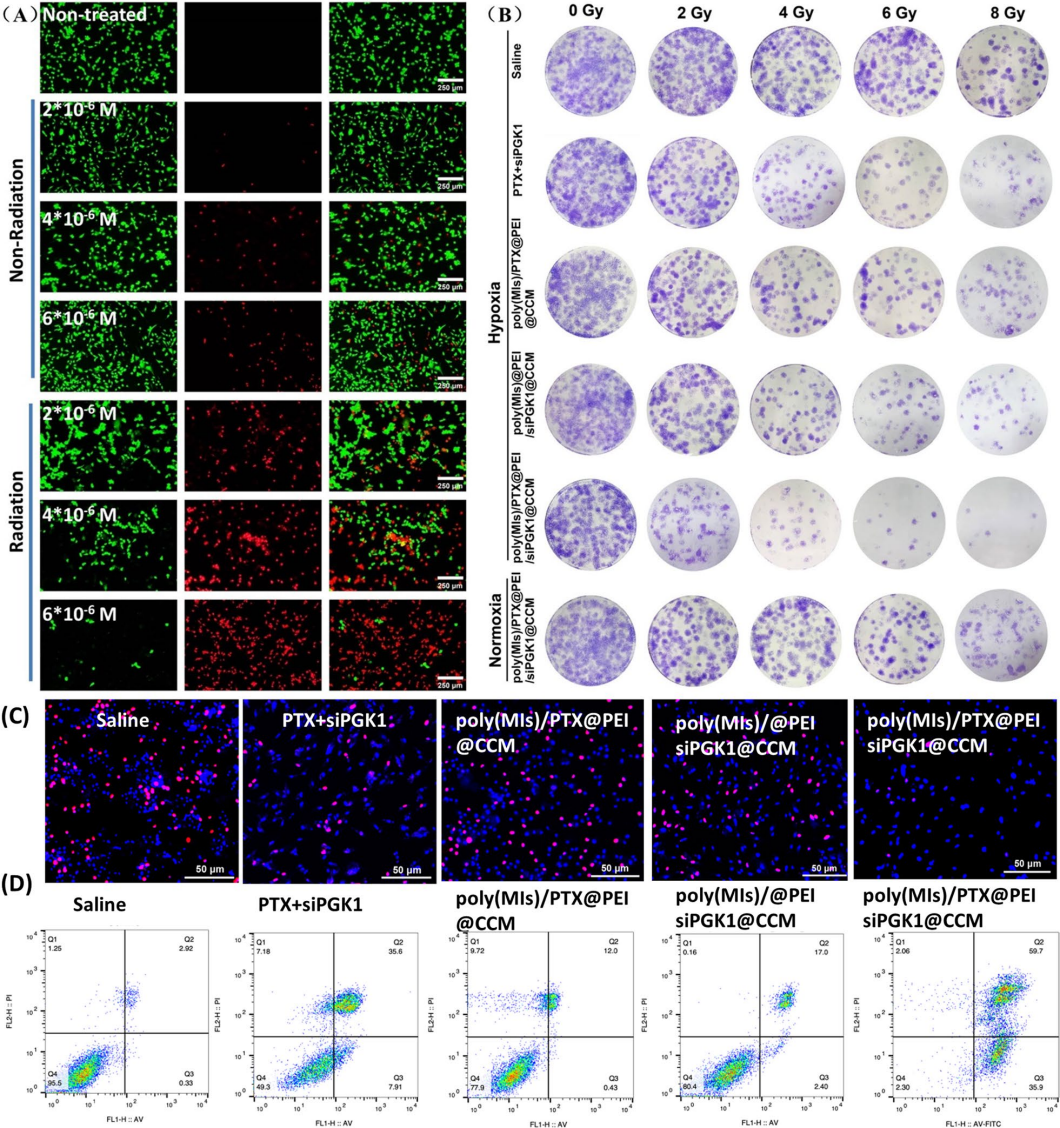
**A** Live/dead cell staining results of U87 cells costained with calcein-AM (live cells, green) and PI (dead cells, red) after hypoxia-responsive activated poly(MIs)/PTX@PEI/siPGK1@CCM treatment at various concentrations. **B** Representative images of the clonogenic survival assays of U87 cells cultured with different formulated micelles under hypoxic conditions (pO_2_: 2%) following treatment with 0, 2, 4, 6, or 8 Gy. **C** EdU-positive cells indicating actively dividing cells. **D** Apoptosis assay of U87MG cells after different treatments and were analyzed using flow cytometry

The apoptosis efficiency of PGK1-mediated chemotherapy was determined by CCK-8 assay (Additional file 1: Fig. S9), and the corresponding chemo/radiotherapy was then evaluated by live/dead cell staining assay (Fig. 4A). Generally, U87MG cell necrosis (stained with propidium iodide) was more bright red in the radiation-treated groups than in the groups without radiation, which demonstrated that radiation was an efficient tool to destroy tumor cells. On the other hand, the dead cells were varied by changing the concentration of poly(MIs)/PTX@PEI/siPGK1@CCM from 2*10^–6^ M to 6*10^–6^ M, indicating that it caused dose-dependent apoptosis and that the concentration of 6*10^–6^ M was efficient in the combination of chemo/radiotherapy. In addition, to further evaluate the efficiency of PGK1-mediated chemo/radiotherapy, a colony forming assay was conducted (Fig. 4B). Treatment with synergistic chemo/radiotherapy in hypoxic tumor cells mediated by PGK1 protein downregulation resulted in a much greater inhibition of colony formation than did the other treatments at the same radiation dose. The inhibitory effects were significantly enhanced with increasing radiation doses from 2 to 8 Gy, and colony formation was almost completely inhibited when the dose was larger than 6 Gy, which was similar to cell cytotoxicity assays (Additional file 1: Fig. S10) and demonstrated an efficient RT-induced apoptosis. The anticancer mechanisms of poly(MIs)/PTX@PEI/siPGK1@CCM RNAi therapy contributed to X-ray-induced DNA damage and poly(MIs)-induced DNA damage fixation. PGK1 protein degradation has been recognized as the major role that sensitizes PTX-induced chemical damage and X-ray-induced ionizing radiation injury by γ-H2AX upregulation (Additional file 1: Fig. S11). This phenomenon was further demonstrated by EdU assays (Fig. 4C), which showed significant effects on DNA duplication. After annexin V/PI double staining, cell apoptosis via different treatments was then captured by flow cytometry (Fig. 4D). The order of total cell apoptosis was consistent with the results of a previous analysis. Taken together, these data revealed that chemotherapy or PGK1 knockdown in combination with radiotherapy independently induced cell death. Furthermore, the synergistic antitumor effect of PGK1-mediated chemo/radiotherapy was predominantly more effective than single treatments.

### In vivo biodistribution

**Fig. 5:**
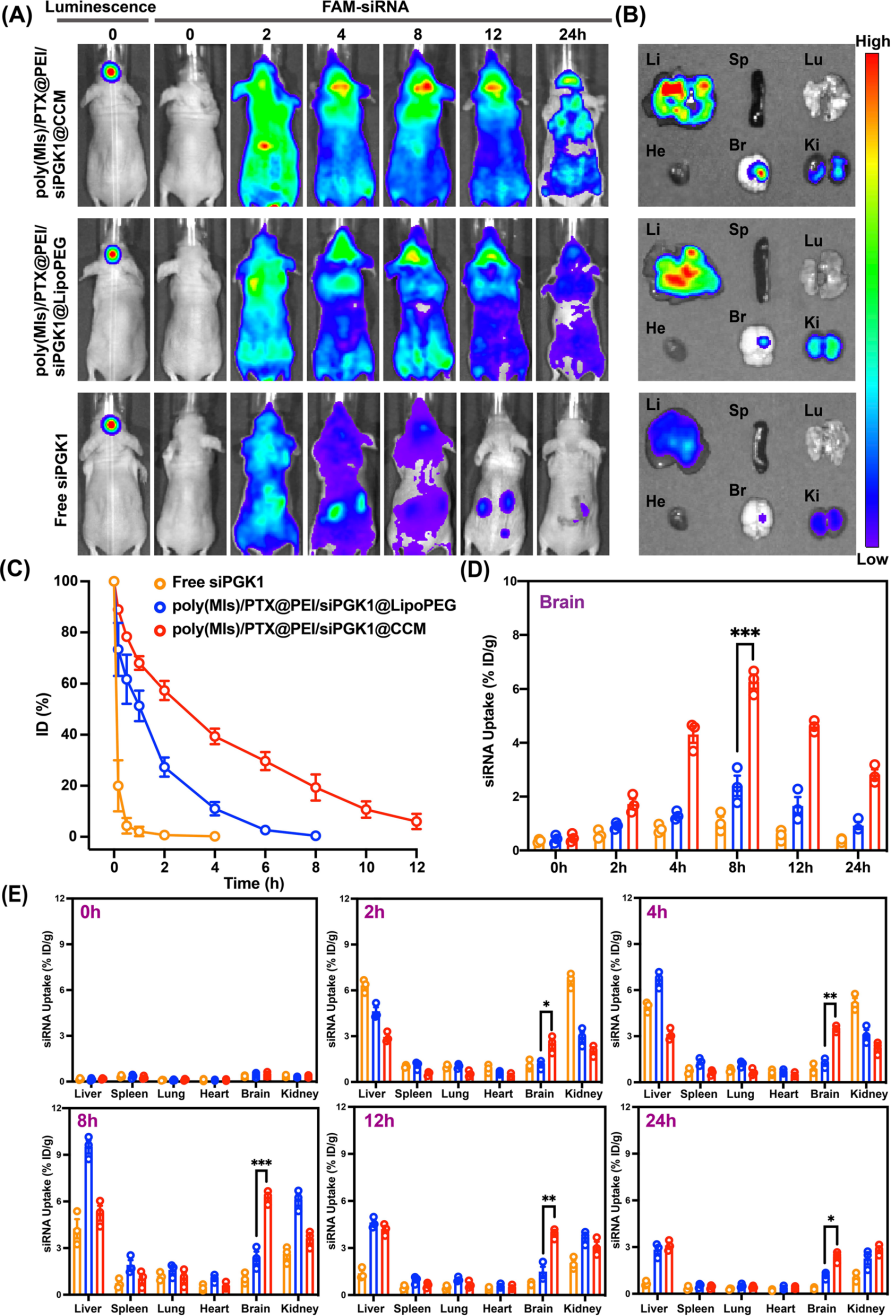
**A** Real-time fluorescence imaging of mice treated with (1) poly(MIs)/PTX@PEI/siPGK1@CCM, (2) poly(MIs)/PTX@PEI/siPGK1@LipoPEG and (3) Free siPGK1, after 20 days of tumor cell inoculation. **B** Ex vivo fluorescence images of major organs dissected from mice injected with poly(MIs)/PTX@PEI/siPGK1@CCM, poly(MIs)/PTX@PEI/siPGK1@LipoPEG and Free siPGK1 (2.3 mg siRNA equiv/kg) at 8 h post injection. H, Li, Sp, Lu, Ki, and Br represent heart, liver, spleen, lung, kidney, and brain, respectively. (**C**) Blood retention kinetics of the free siPGK1, poly(MIs)/PTX@PEI/siPGK1@LipoPEG and poly(MIs)/PTX@PEI/siPGK1@CCM in tumor-free BALB/C mice. **D** Quantitative analysis of siPGK1 in brain tissues at different time points. **E** Quantitative biodistribution of the different treatments in the brains and major organs of the mice at 0 h, 2 h, 4 h, 8 h, 12 h and 24 h. Data are shown as mean ± SD (n = 3). (*p < 0.05, **p < 0.01, ***p < 0.001)

To further study in vivo pharmacokinetics, the blood distribution of poly(MIs)/PTX@PEI/siPGK1@CCM with time after a single intravenous injection was investigated (Fig. 5C). Obviously, free siPGK1 was rapidly cleared from the bloodstream. Conversely, poly(MIs)/PTX@PEI/siPGK1@CCM and poly(MIs)/PTX@PEI/siPGK1@Lipo- PEG exhibited extended elimination half-lives (t1/2) of approximately 3.5 h and 1.8 h, respectively, both of which were much longer than those of free siPGK1. Furthermore, poly(MIs)/PTX@PEI/siPGK1@CCM showed an ~ twofold longer blood circulation time than poly(MIs)/PTX@PEI/siPGK1@LipoPEG, which demonstrated that the CCM layer extended the half-lives more efficiently than the PEG shell, effectively avoiding the immune system clearance. To further investigate the imaging performances of these biomimetic nanoparticles in the glioma, the tumor cell inoculation in the brain of nude mice for 20 days was conducted. The fluorescence signals appeared at 2 h, reached the highest at 8 h and lasted for at least 24 h after intravenous administration (Fig. 5A). The accumulation of nanoparticles in glioma cells was superior to both poly(MIs)/PTX@PEI/siPGK1@LipoPEG and Free siPGK1 at all investigated time points (2, 4, 8, 12 and 24 h), which exhibited CCM coating-specific targeting and specific accumulation in GBM tumors. More importantly, the fluorescence signal of poly(MIs)/PTX@PEI/siPGK1 @CCM in the brain greatly decreased at 24 h compared with that of poly(MIs)/PTX@PEI/siPGK1@LipoPEG, which maintained a long fluorescence signal in the brain for up to 24 h. This difference was attributed to the efficient siPGK1 release of poly(MIs)/PTX@PEI/siPGK1@CCM after accumulation, which was triggered by hypoxic brain tissue. Furthermore, ex vivo imaging of the excised brain demonstrated that the fluorescence was localized in orthotopic brain tumor tissue instead of other normal tissues (Fig. 5B), which demonstrated that poly(MIs)/PTX@PEI/siPGK1@CCM could efficiently cross BBB permeation and accumulate in orthotopic tumors. In addition, the quantitative analysis showed that the concentration of poly(MIs)/PTX@PEI/siPGK1@CCM at 2 h, 4 h, 8 h, 12 h and 24 h was much higher than that of the poly(MIs)/PTX@PEI/siPGK1@LipoPEG group (Fig. 5D), which was consistent with previous fluorescence imaging analysis that further demonstrated that poly(MIs)/PTX@PEI/siPGK1@CCM had better brain tumor targeting ability than the poly(MIs)/PTX@PEI/siPGK1@LipoPEG formulation. The quantitative distribution profiles of both nanoformulations in major organs were measured (Fig. 5E), and these data indicated that the liver and kidney were the main sites of accumulation. Moreover, in the GBM tissue of mice injected with poly(MIs)/PTX@PEI/siPGK1@CCM, the effluent green fluorescence of FAM-siPGK1 overlapped with the DAPI-stained cell nuclei. In contrast, the poly(MIs)/PTX@PEI/siPGK1@LipoPEG group showed less fluorescence intensity (Additional file 1: Fig. S12), which revealed that poly(MIs)/PTX@PEI/siPGK1@CCM could cross the BBB and deliver the siRNA into GBM and provided the basis for the next validation of our effective RNAi therapy in vivo.

### In vivo anticancer effect on U87MG tumor-bearing mice

**Fig. 6:**
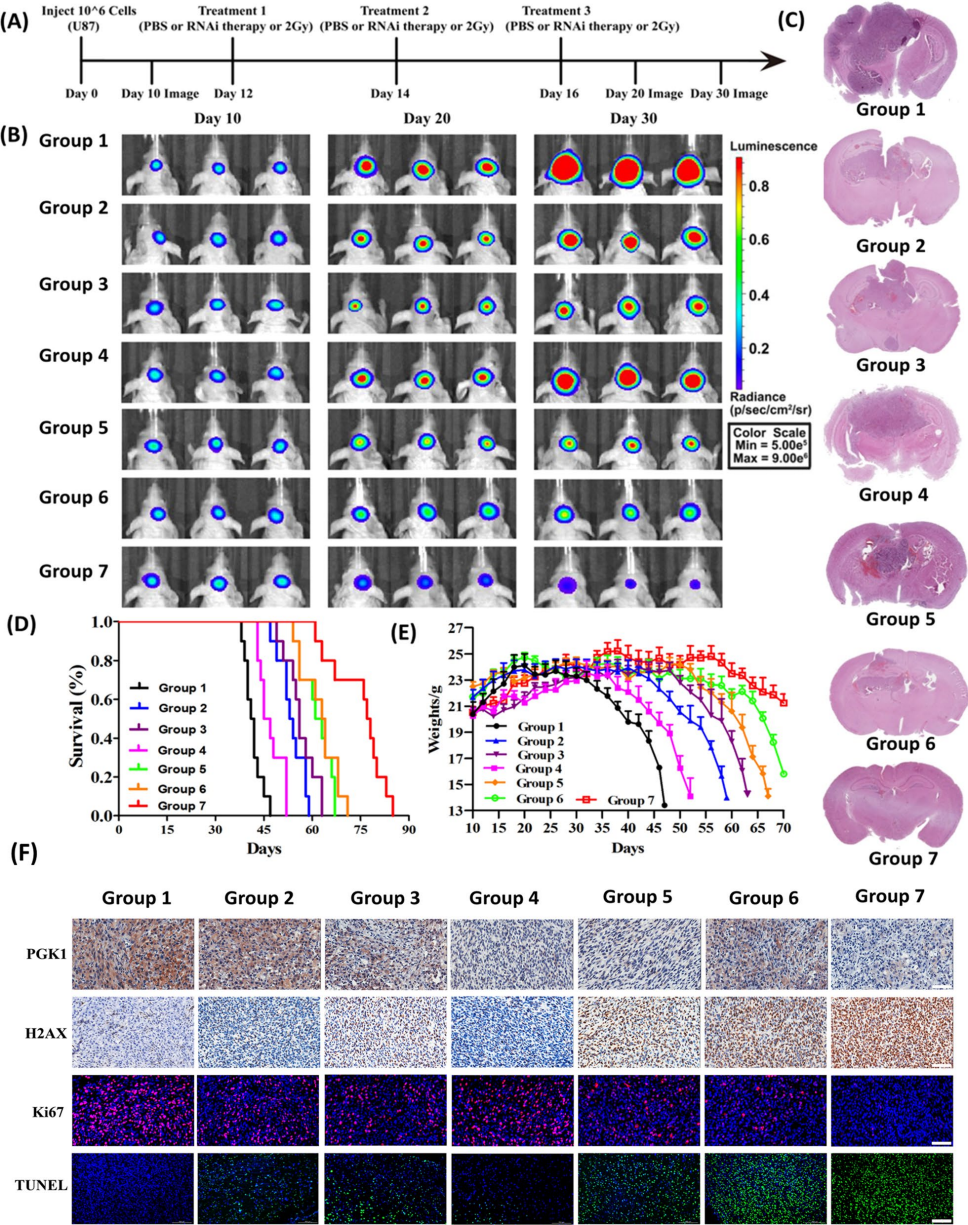
**A** Schematic protocol of animal experiments. **B** In vivo efficacy in the orthotopic U87MG-luc mouse model by bioluminescence signal (saline, saline + RT, free PTX + naked siPGK1 + RT, poly(MIs)/PTX@PEI/siPGK1@CCM (without RT), poly(MIs)@PEI/siPGK1@CCM + RT, poly(MIs)/PTX@PEI@CCM + RT, poly(MIs)/PTX@PEI/siPGK1@CCM + RT at doses of 1.8 mg/kg PTX and 2.3 mg/kg siPGK1) on days 10, 20 and 30 with 2 Gy RT. **C** Typical H&E staining images of tumors (coronal staining). **D** Kaplan–Meier survival curves for the mice (n = 10) after different treatment strategies. **E** Body weight change in mice. The data are presented as the mean ± SDs. **F** Tumor slices excised from nude mice bearing orthotopic U87MG-luc human glioblastoma tumors following treatment using PGK1, rH2AX, TUNEL and proliferation (Ki67) staining

Inspired by the in vitro investigation, to assess the antitumor efficacy of poly(MIs)/PTX@PEI/siPGK1@CCM in vivo, we established U87MG glioblastoma orthotopic xenograft models with Balb/c mice. Briefly, as depicted in our experiments (Fig. 6A), the successful presence of brain glioblastoma in mice was confirmed at 10 days using an in vivo animal imaging system by luciferase detection, and different treatment strategies were carried out at day 12. The tumor growth of orthotopic glioblastoma cells after different treatment strategies was easily monitored by injecting luciferin followed by in vivo bioluminescence imaging. Obviously, the PBS-treated groups (group 1) showed no effect on orthotopic glioblastoma proliferation, which presented a much larger tumor volume at day 30 than at day 10 (Fig. 6B). In general, after successive treatment, various other strategy-treated groups showed a noticeable difference in tumor suppression. Notably, the enhanced antitumor effect of poly(MIs)/PTX@PEI/siPGK1@CCM + RT (group 7) was confirmed by the lower bioluminescence intensity compared with that of the other groups. More importantly, the mice treated with only nanoformulation-based siPGK1 therapy (group 5) or nanoformulation-based chemotherapy (group 6) exhibited tumor suppression activity to different degrees, suggesting that PGK1 mediated chemotherapy and radiotherapy and presented a synergetic effect. Clearly, CCM had an important role in tumor recognition and deep penetration that was beneficial for subsequent PGK1 downregulation, PTX-induced chemotherapy and radiotherapy, which was effectively demonstrated by their significant reduction in U87MG-Luc bioluminescence. Furthermore, the tumor inhibition of all groups was visibly characterized by H&E staining (Fig. 6C), and the results of the histologic analysis were consistent with the Luc-bioluminescence results. To further estimate the antitumor efficacy, the median survival times of the U87-Luci-bearing mice were recorded and monitored (Fig. 6D**)**. Notably, the mice treated with poly(MIs)/PTX@PEI/siPGK1@CCM + RT had a long median survival time of 77.5 days. In contrast, the mice treated with poly(MIs)/PTX@PEI/siPGK1@CCM without RT (group 4) had a relatively short median survival time of 46 days, suggesting that radiotherapy was of great importance to tumor inhibition, ascribing to that hypoxic tumor environment was always occurred in the deep tissue or solid tumor center, which limited the efficient disassociation of our hypoxia-responsive poly(MIs)/PTX@PEI/siPGK1@CCM. To further assess the in vivo safety, body weight was recorded in our treatment (Fig. 6E). All results showed that the poly(MIs)/PTX@PEI/siPGK1@CCM nanomedicine had high biocompatibility in mice with implanted orthotopic brain tumors. After poly(MIs)/PTX@PEI/siPGK1@CCM + RT treatment, the body weights of mice had no obvious decrease that presented good safety in vivo and indicated superior tumor inhibition compared with that in the PBS group, of which the mice lost weight rapidly. Furthermore, immunofluorescence and immunohistochemistry (IHC) images showed that poly(MIs)/PTX@PEI/siPGK1@CCM + RT (group 7) exhibited the highest level of tumor cell apoptosis and the lowest level of tumor cell proliferation (Fig. 6F). Remarkably, obvious PGK1 reduction was observed for tumor slides taken from all groups treated with poly(MIs)/PTX@PEI/siPGK1@CCM, and this phenomenon indicated effective siPGK1 protein downregulation. Subsequently, tumor tissue, including heart, liver, spleen, lung and kidney, from mice treated with different strategies was further analyzed (Additional file 1: Fig. S13), and tumor tissue from mice treated with poly(MIs)/PTX@PEI/siPGK1@CCM showed no obvious damage to these major organs. In addition, poly(MIs)/PTX@PEI/siPGK1@CCM had a negligible influence on liver and kidney functions or hematological parameters by blood biochemistry and hematology results (Additional file 1: Fig. S14). On one hand, these results demonstrated that our synthesized poly(MIs)/PTX@PEI/siPGK1@CCM RNAi nanomedicine has promising clinical applications in GBM therapy. On other hand, some limitations still existed in this study and the major one is the use of immune-deficient mice and cell line-derived GBM that could not be representative of patient-GBM.

## Conclusion

In the present study, we successfully established a biomimetic hypoxia-triggered RNAi nanomedicine (poly(MIs)/PTX@PEI/siPGK@CCM) that exhibited superior glioblastoma inhibition in vitro and significantly prolonged survival in vivo without obvious systemic immune clearance. The novel amphiphilic cationic block copolymer poly(MIs)-PEI was successfully synthesized for PTX and siPGK1 delivery with unique hypoxia-triggered properties, which presented efficient knockdown of PGK1 protein that offered enough energy for glioblastoma growth and metastasis. Notably, this RNAi nanomedicine strongly increased tumor accumulation with desirable biocompatibility by self-recognition and tumor-selective “homing” to the homologous tumor. In general, our synthesized RNAi nanomedicine may represent an excellent RNAi strategy for GBM treatment that synergistically enhances sensitivity to chemotherapy and radiotherapy and shows promising clinical application.

## Supplementary Information

**Additional file 1: Figure S1**: Synthetic routes of poly(MIs)-PEI block copolymers by ROP reaction and condensation reaction; **Figure S2**: ^1^H NMR spectra of poly(MIs)-PEI block copolymers; **Figure S3**: FT-IR spectra of poly(MIs)-PEI block copolymers;** Figure S4**: Size distribution of poly(MIs)/PTX@PEI, poly(MIs)/PTX@PEI/siPGK1 and poly(MIs)/PTX@PEI/siPGK1@CCM in PBS; **Figure S5**: Size distribution of poly(MIs)/PTX@PEI/siPGK1@CCM under normoxic and hypoxic conditions; **Figure S6**: The stability poly(MIs)@PEI@CCM, poly(MIs)/PTX@PEI@CCM, poly(MIs)@PEI/siPGK1@CCM and poly(MIs)/PTX@PEI/ siPGK1@CCM NPs were investigated in DMEM containing 10% FBS over seven days; **Figure S7**: Sequential release of siPGK1 and PTX from poly(MIs)/PTX@PEI/siPGK1@CCM; **Figure S8**: Quantitative analysis of cumulative transport of poly(MIs)/PTX@PEI/ siPGK1@CCM at different time points across the bEnd.3 monolayer; **Figure S9**: Cell viability of poly(MIs)/PTX@PEI/siPGK1@CCM with various concentration in normoxic and hypoxic conditions, respectively; **Figure S10**: Cell viability of poly(MIs)/PTX@PEI/siPGK1@CCM with different radiation dose; **Figure S11**: Immunocytochemical analysis of γ-H2AX expressed by U87 cells. Cells were stained with an anti-γ-H2AX antibody (red) and DAPI (blue) after RT; **Figure S12**:Fluorescence images of frozen sections of tumor tissue (at a siPGK1 concentration of 2.3 mg kg^-1^) at different times after injection of poly(MIs)/PTX@PEI/siPGK1@LipoPEG and poly(MIs)/PTX@PEI/siPGK1@CCM. FAM-siPGK1 (green), and cell nuclei (blue); **Figure S13**: Representative tissue sections of mice stained with hematoxylin and eosin (H&E) after 30 days of different treatments; **Figure S14**: Results analysis of liver and kidney functions by blood biochemistry after treatment with poly(MIs)/PTX@PEI/siPGK1@CCM. The Supporting Information is available free of charge on the website.

## Data Availability

The datasets and materials used in the study are available from the corresponding author.
