# Supplementary material for: Biomimetic hypoxia-triggered RNAi nanomedicine for synergistically mediating chemo/radiotherapy of glioblastoma
Source: J Nanobiotechnology. 2023 Jul 5;21:210. doi: 10.1186/s12951-023-01960-w (PMC10324258; doi:10.1186/s12951-023-01960-w)
Supplement: Supplementary file 1 — Additional file 1: Figure S1: Synthetic routes of poly(MIs)-PEI block copolymers by ROP reaction and condensation reaction; Figure S2: 1H NMR spectra of poly(MIs)-PEI block copolymers; Figure S3: FT-IR spectra of poly(MIs)-PEI block copolymers; Figure S4: Size distribution of poly(MIs)/PTX@PEI, poly(MIs)/PTX@PEI/siPGK1 and poly(MIs)/PTX@PEI/siPGK1@CCM in PBS; Figure S5: Size distribution of poly(MIs)/PTX@PEI/siPGK1@CCM under normoxic and hypoxic conditions; Figure S6: The stability poly(MIs)@PEI@CCM, poly(MIs)/PTX@PEI@CCM, poly(MIs)@PEI/siPGK1@CCM and poly(MIs)/PTX@PEI/ siPGK1@CCM NPs were investigated in DMEM containing 10% FBS over seven days; Figure S7: Sequential release of siPGK1 and PTX from poly(MIs)/PTX@PEI/siPGK1@CCM; Figure S8: Quantitative analysis of cumulative transport of poly(MIs)/PTX@PEI/ siPGK1@CCM at different time points across the bEnd.3 monolayer; Figure S9: Cell viability of poly(MIs)/PTX@PEI/siPGK1@CCM with various concentration in normoxic and hypoxic conditions, respectively; Figure S10: Cell viability of poly(MIs)/PTX@PEI/siPGK1@CCM with different radiation dose; Figure S11: Immunocytochemical analysis of γ-H2AX expressed by U87 cells. Cells were stained with an anti-γ-H2AX antibody (red) and DAPI (blue) after RT; Figure S12:Fluorescence images of frozen sections of tumor tissue (at a siPGK1 concentration of 2.3 mg kg-1) at different times after injection of poly(MIs)/PTX@PEI/siPGK1@LipoPEG and poly(MIs)/PTX@PEI/siPGK1@CCM. FAM-siPGK1 (green), and cell nuclei (blue); Figure S13: Representative tissue sections of mice stained with hematoxylin and eosin (H&E) after 30 days of different treatments; Figure S14: Results analysis of liver and kidney functions by blood biochemistry after treatment with poly(MIs)/PTX@PEI/siPGK1@CCM. The Supporting Information is available free of charge on the website. [file 12951_2023_1960_MOESM1_ESM.docx]

**Additional file Information**

Biomimetic hypoxia-triggered RNAi nanomedicine for synergistically mediating chemo/radiotherapy of glioblastoma

Zhen Wang^a‡^, Xianglong Tang^b,c‡*^, Mengjie Zhao^b,c‡^, Yong Xiao^a‡^, Yiding Zhang^a^, Yuyang Liu^a^, Chunfa Qian^a^, Yan-dong Xie ^a^, Yong Liu^a^, Yuanjie Zou^a^, Kun Yang^a^* and Hongyi Liu^a,b,c^*

^a^ Department of Neurosurgery, the Affiliated Brain Hospital with Nanjing Medical University, Fourth Clinical College of Nanjing Medical University, Nanjing 210029, China

^b^ Department of Neuro-Psychiatric Institute, the Affiliated Brain Hospital with Nanjing Medical University, Nanjing 210029, China

^c^ Institute of Neuro-science, Nanjing Medical University, Nanjing 210029, China

* Correspondence should be addressed to H.Y. Liu (njnkyylhy@163.com) or K.Y. ([yk_nj@hotmail.com](mailto:yk_nj@hotmail.com)) or X.L.Tang ([xltang0326@163.com](mailto:xltang0326@163.com))

(1)

(2)

(3)

(4)

**Figure S1.** Synthetic routes of poly(MIs)-PEI block copolymers by ROP reaction and condensation reaction.


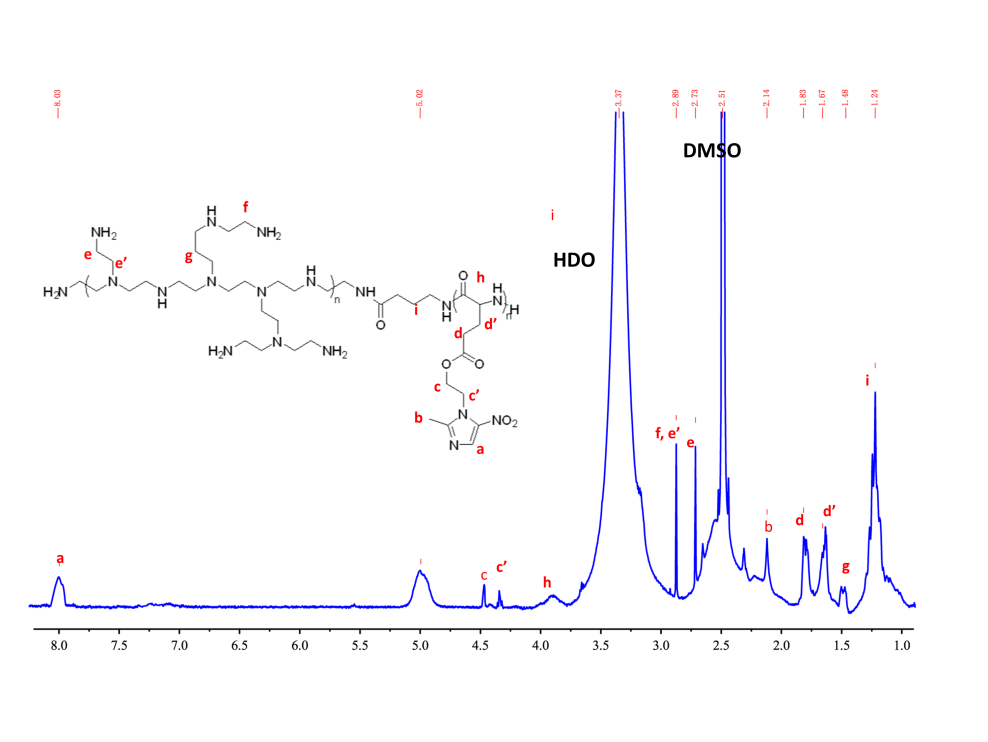


**Figure S2.** ^1^H NMR spectra of poly(MIs)-PEI block copolymers by D_2_O exchange of active hydrogen (D_2_O/DMSO-d6, 400 MHz).


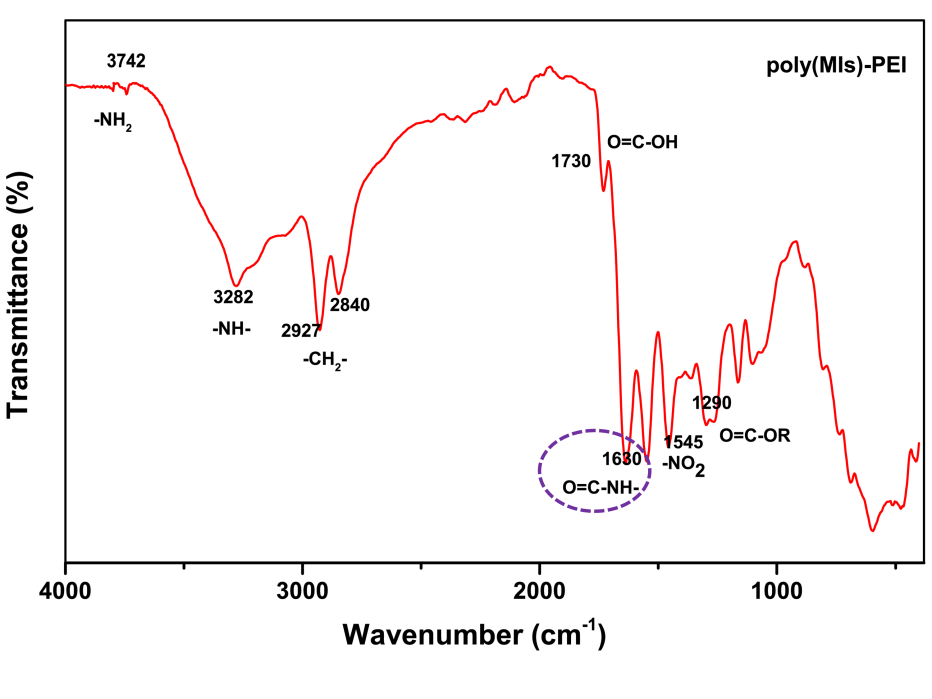


**Figure S3.** FT-IR spectra of poly(MIs)-PEI block copolymers.


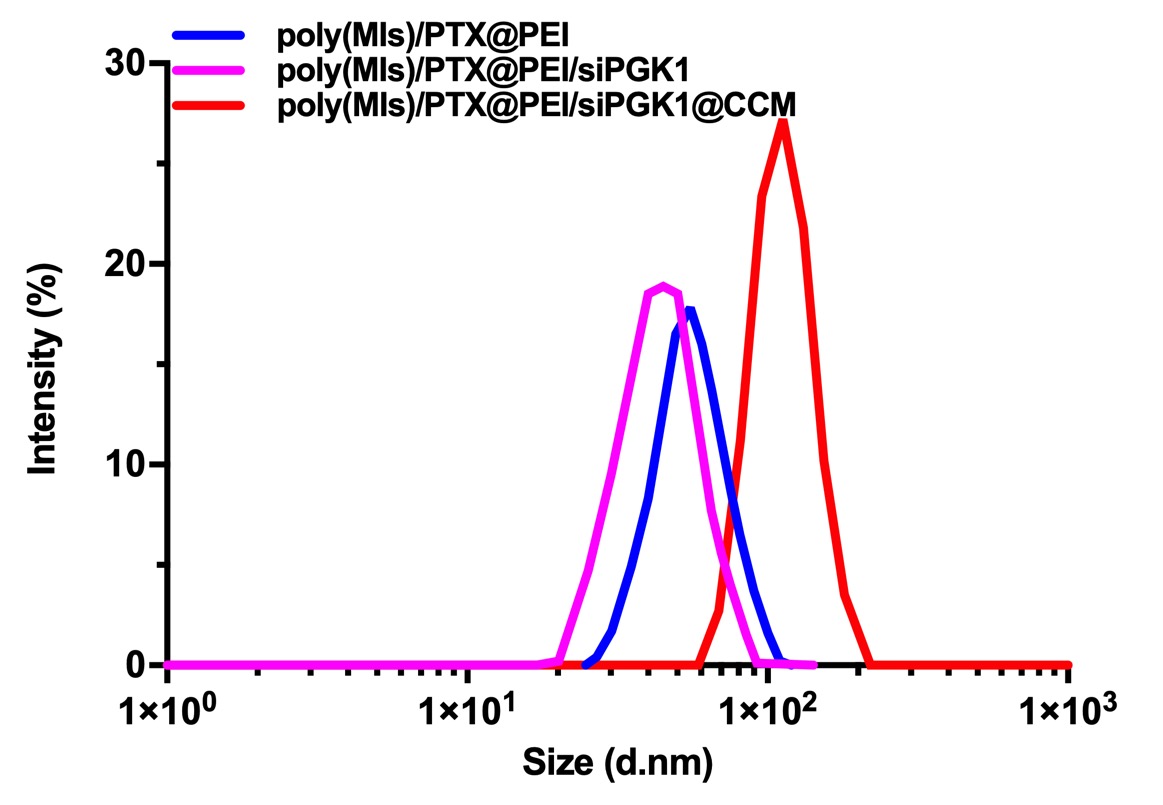


**Figure S4.** Size distribution of poly(MIs)/PTX@PEI, poly(MIs)/PTX@PEI/siPGK1 and poly(MIs)/PTX@PEI/siPGK1@CCM in PBS.


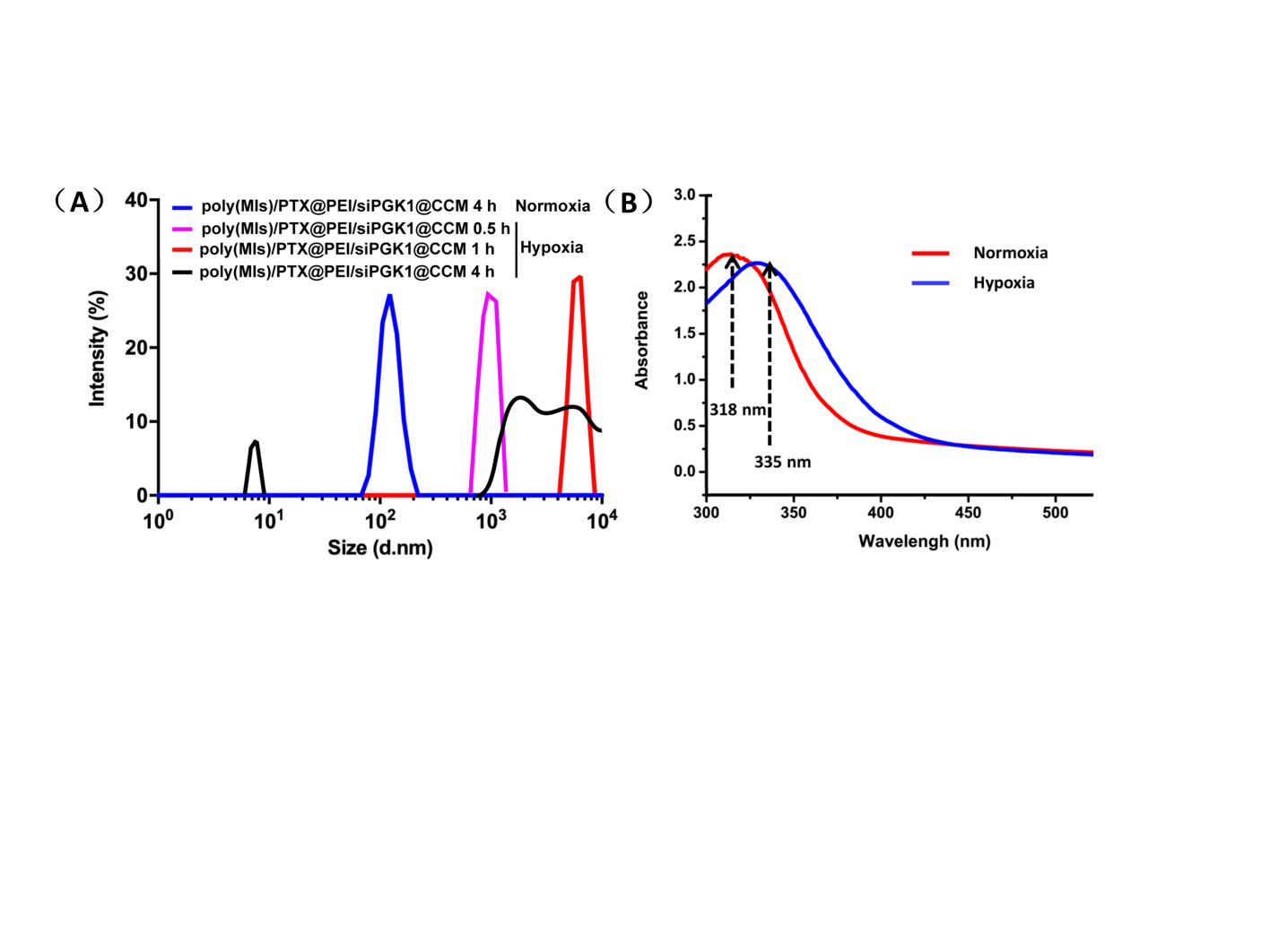


**Figure S5.** (A) Size distribution of poly(MIs)/PTX@PEI/siPGK1@CCM under normoxic and hypoxic conditions. (B) UV-Vis absorption spectra of poly(MIs) under normoxia and hypoxia.


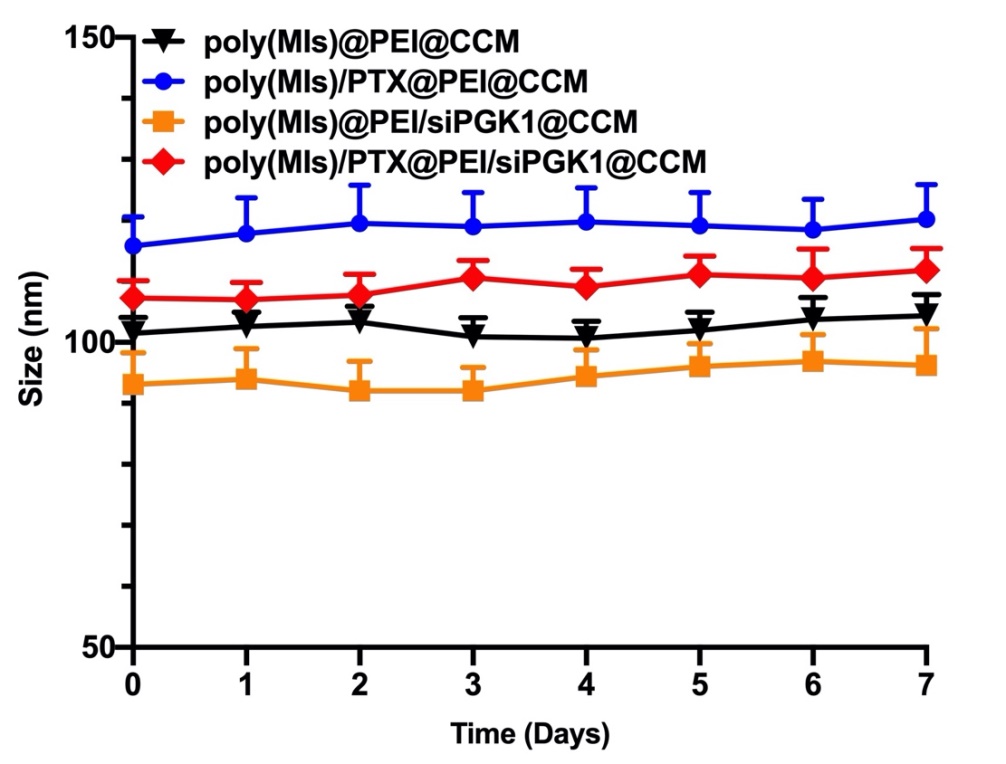


**Figure S6.** The stability of poly(MIs)@PEI@CCM, poly(MIs)/PTX@PEI@CCM, poly(MIs)@PEI/siPGK1@CCM and poly(MIs)/PTX@PEI/siPGK1@CCM NPs were investigated in DMEM containing 10% FBS over seven days. Data are shown as mean ± SD (n=3).


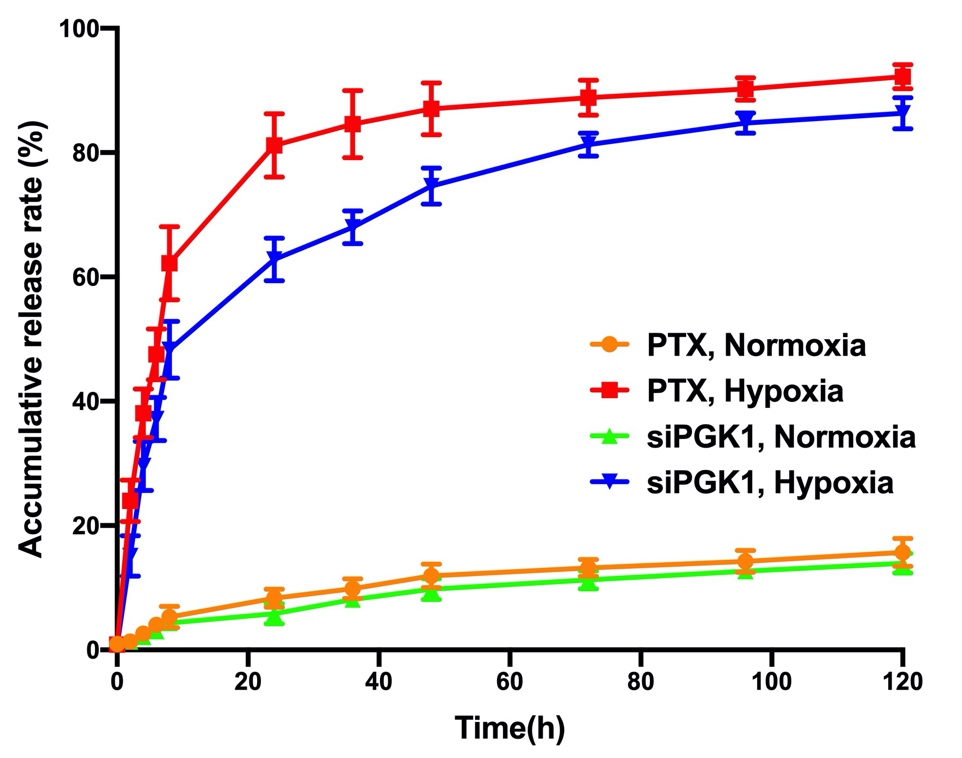


**Figure** **S7**. Sequential release of siPGK1 and PTX from poly(MIs)/PTX@PEI/ siPGK1@CCM. Data are shown as mean ± SD (n=3).


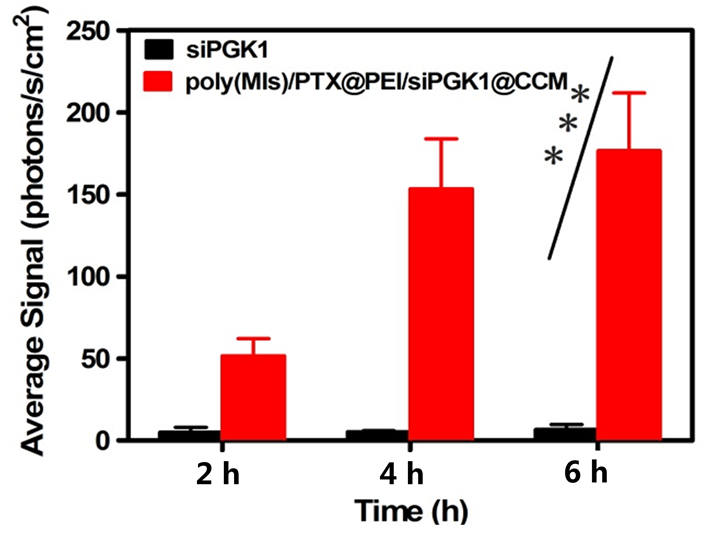


**Figure S8.** Quantitative analysis of cumulative transport of poly(MIs)/PTX@PEI/ siPGK1@CCM at different time points across the bEnd.3 monolayer. Data are shown as mean ± SD (n=3).


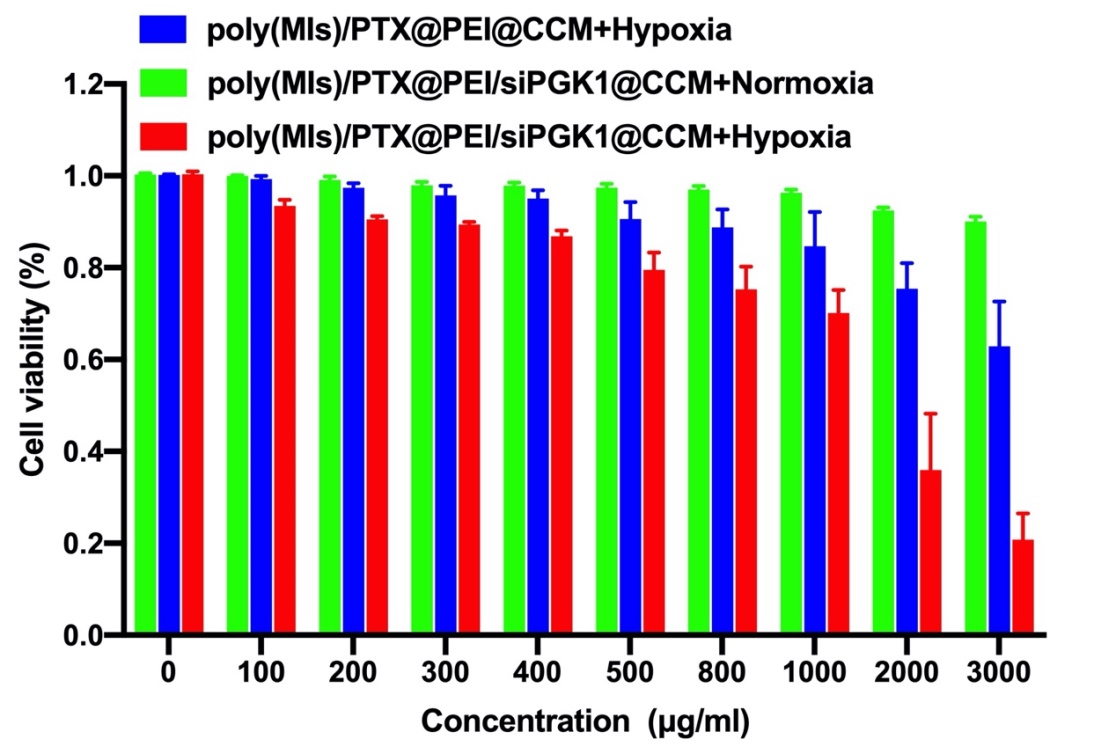


**Figure S9.** Cell viability of poly(MIs)/PTX@PEI/siPGK1@CCM with various concentration in normoxia and hypoxia, respectively. Data are shown as mean ± SD (n=3).


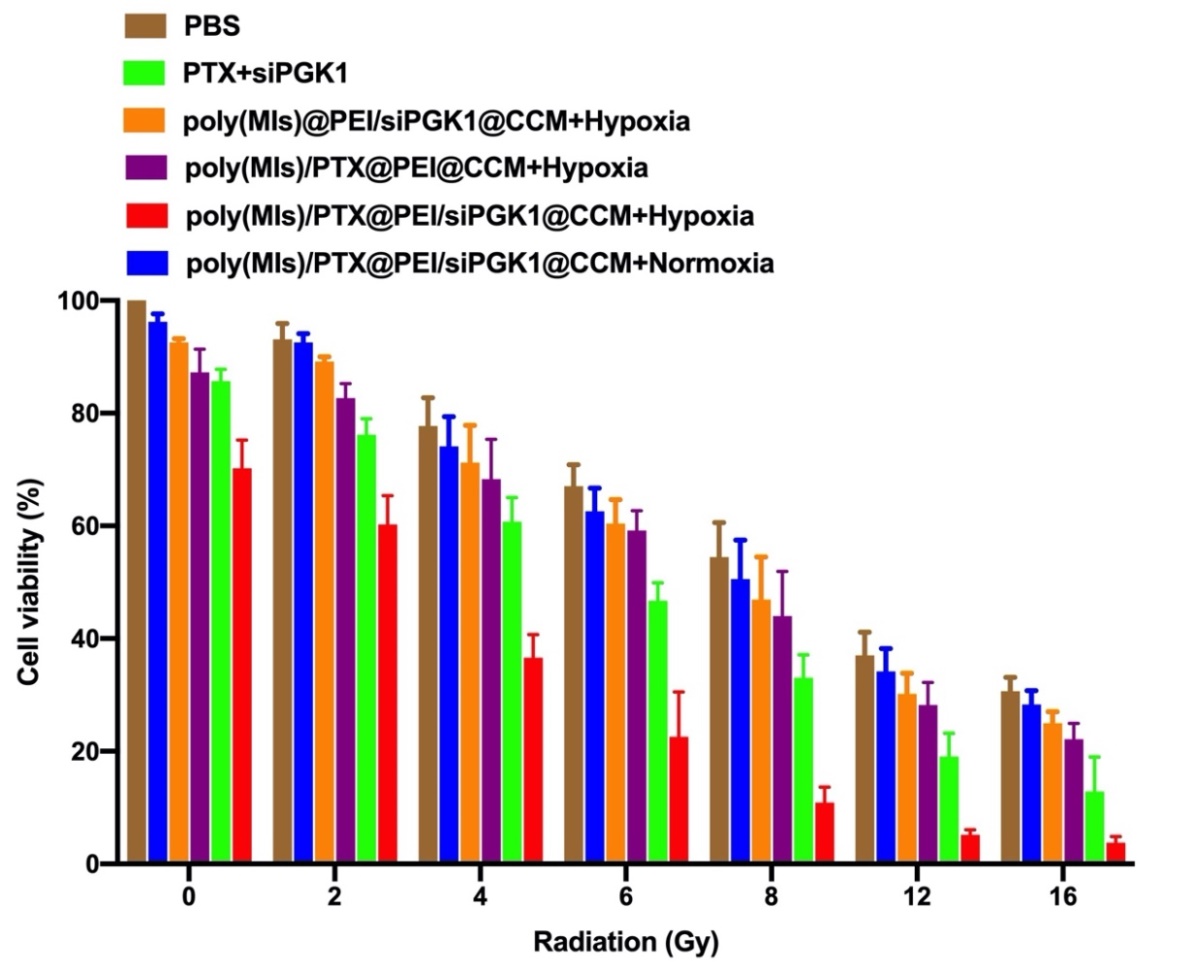


**Figure S10.** Cell viability of poly(MIs)/PTX@PEI/siPGK1@CCM with different radiation dose. Data are shown as mean ± SD (n=3).


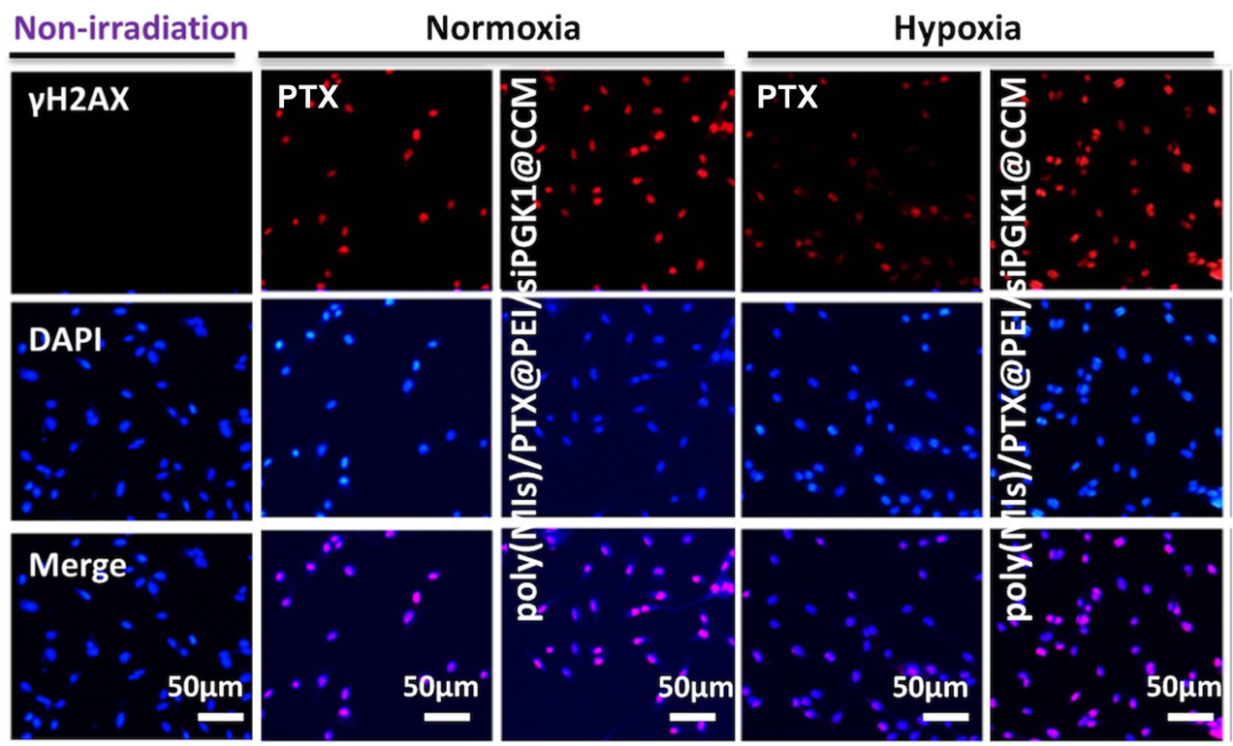


**Figure S11.** Immunocytochemical analysis of γ-H2AX expressed by U87 cells. Cells were stained with an anti-γ-H2AX antibody (red) and DAPI (blue) 24 h after RT


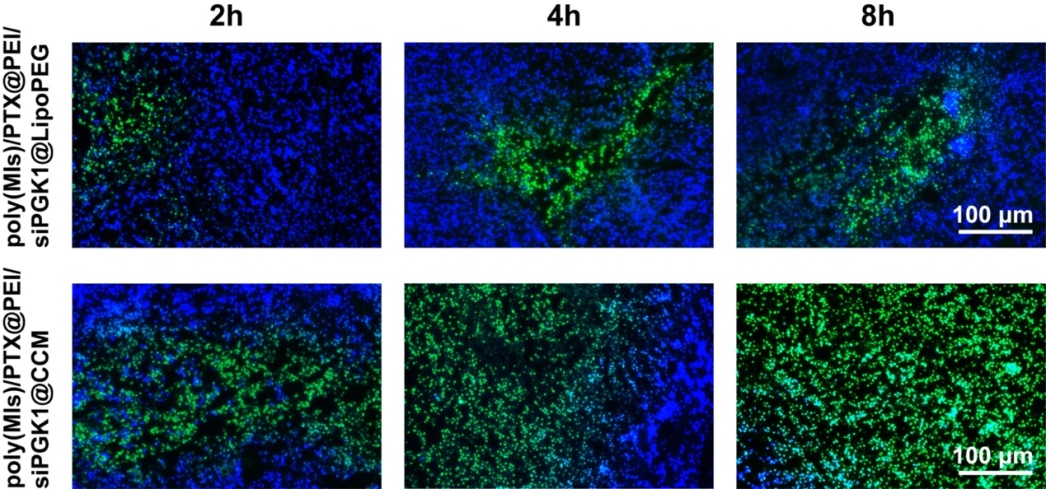


**Figure S12.** Fluorescence images of frozen sections of tumor tissue (at a siPGK1 concentration of 2.3 mg kg^-1^) at different times after injection of poly(MIs)/PTX@PEI/siPGK1@LipoPEG and poly(MIs)/PTX@PEI/siPGK1@CCM. FAM-siPGK1 (green), and cell nuclei (blue). (Scale bar: 100 μm)


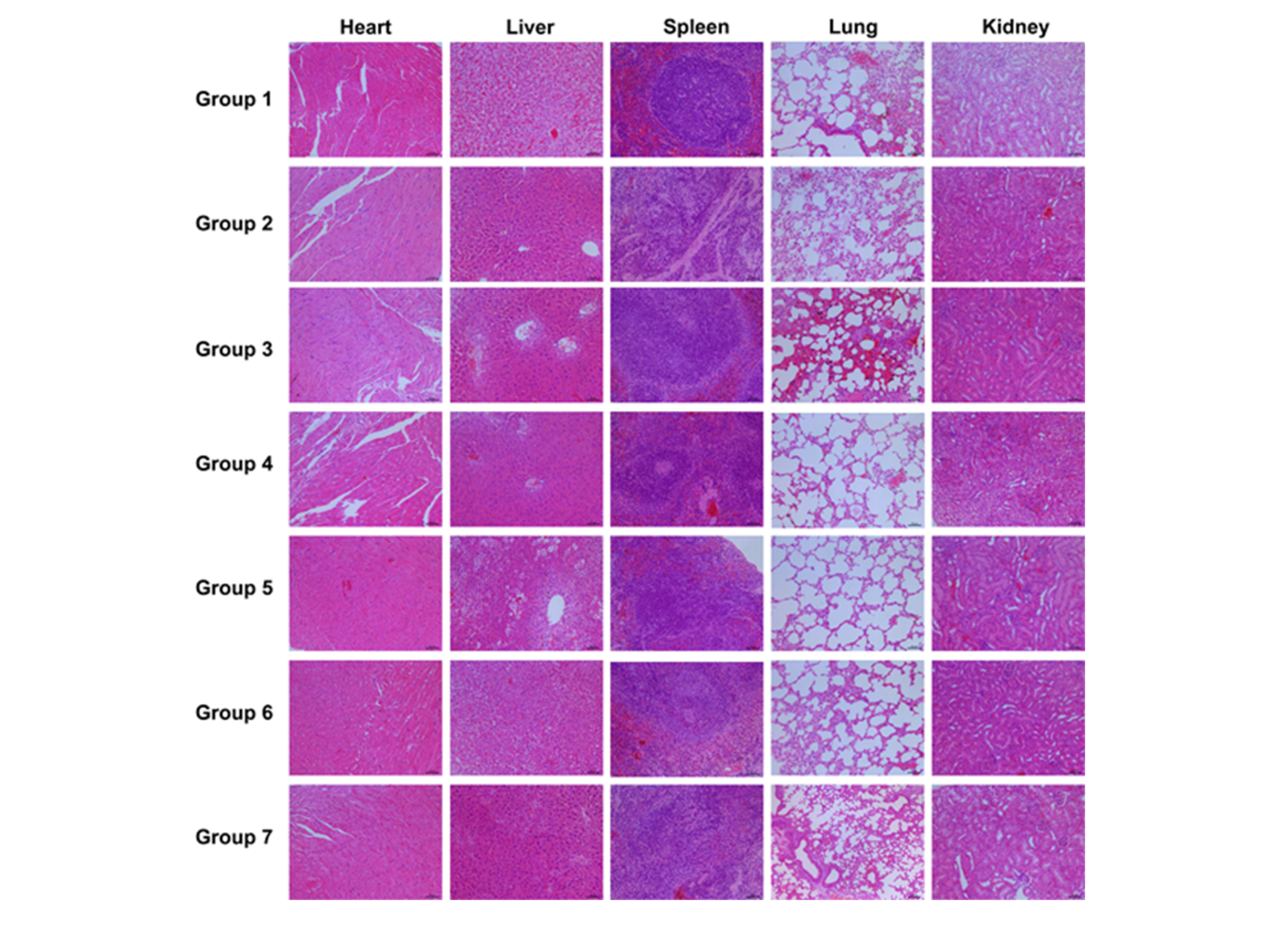


**Figure S13.** Representative tissue sections of mice stained with hematoxylin and eosin (H&E) after 30 days of different treatments.


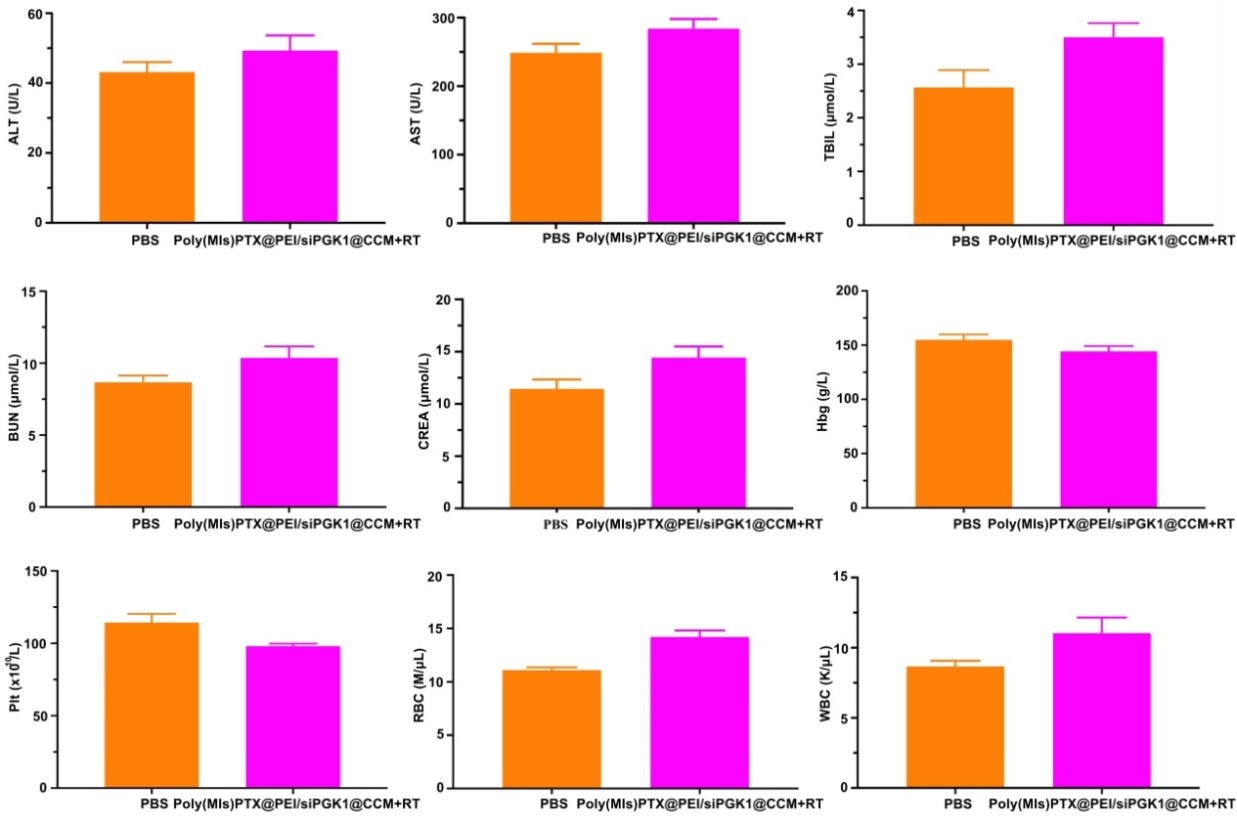


**Figure S14.** Results analysis of liver and kidney functions by blood biochemistry after treatment with poly(MIs)/PTX@PEI/siPGK1@CCM.
